# Assessing the development of primary English education based on CIPP model—a case study from primary schools in China

**DOI:** 10.3389/fpsyg.2024.1273860

**Published:** 2024-06-14

**Authors:** Xiao Chang, Zhe Wang

**Affiliations:** ^1^College of Foreign Languages, Changsha Normal University, Changsha, China; ^2^School of Mathematics and Science, Hebei GEO University, Shijiazhuang, China; ^3^Department of Statistics, College of Natural Science, Jeonbuk National University, Jeonju-si, Republic of Korea

**Keywords:** primary English, English education, primary English education (PEE), sustainable development, CIPP model

## Abstract

This article examines the development of primary English education from teachers’ and students’ perspectives; this is done by investigating environment, curriculum and teacher and students’ information. The study was carried out with 42 primary English teachers and 404 primary students from 90 urban and rural primary schools in 13 regions in the Hunan Province, China, the participants engaged in the questionnaire tasks to investigate the present status and problems with primary English education in the Hunan Province. Based on the assessment indicators through CIPP model, combining educational theory and sustainable development theory, the present study develops a primary school English education evaluation model and conducts a practical investigation of primary school English education in the Hunan Province, based on the established model. The findings indicate that the quality of teachers has improved, teaching methods and teaching equipment have become diversified, and student interests and English proficiencies have increased. However, there are still deficiencies in the implementation of Primary English Education in the Hunan Province. For instance, certain schools improperly implement national policies, resulting in imbalanced education. Additionally, educational inequality persists due to disparities in family economic status and importance. Moreover, regional, ideological, and management factors contribute to uneven allocation of educational resources. Furthermore, significant disparities exist between urban and rural areas in terms of teacher qualifications, teaching quality, and school operating hours. The article proposes enhancing awareness of sustainable development, strengthening supervision, and seeking educational and policy support to facilitate the sustainable development of primary English education.

## 1 Introduction

The English language has become the cornerstone for global communication and comprehension, regardless of whether it is in English-speaking or non-English-speaking nations (Zhang, 2009, 2021; Emery, 2012; Taylor et al., 2015). The optimal timing for commencing English language acquisition remains a subject of intense debate (Fan, 2007); however, primary English education (PEE) has emerged as an integral component of education in various countries. It is not surprising that research on primary school education is extensive in numerous countries such as Australia, New Zealand (Taylor et al., 2015), Vietnam (Nguyen, 2011), China (Hu, 2007), Mexico (Sayer, 2015), Malaysia (Azman, 2016), Nepal (Sah, 2022), among others. Consequently, within this body of literature, the study on PEE has also emerged as a pivotal component of English education research.

Much of the research on PEE has investigated the educational policies of English (Hoa and Tuan, 2007; Hu, 2007; Nguyen, 2011; Hou, 2012; Hoo, 2013; Sulistiyo et al., 2020; Sah, 2022). The case study conducted by Nguyen (2011) examined the curricula of primary education in Vietnam, focusing on private and public schools, as well as the policies implemented in the country. One notable policy explored was the introduction of English teaching starting from grade 3. In Nepal, English has been incorporated as a mandatory subject from grades 1 to 5 since 2004. Moreover, primary schools not only provide English classes but also deliver other subjects in both English and Nepali languages. The significance given to English can be observed through the increase in annual learning hours from 128 h in grades 1–3 to 160 h in senior grades. Furthermore, this emphasis is evident in the teaching objectives that explicitly emphasize the necessity for students to have comprehensive exposure to English language, offer them opportunities for learning within and beyond campus boundaries, and foster their proficiency and enthusiasm for effective communication and written expression (Sah, 2022). The aforementioned studies focused on teaching arrangements and objectives in Vietnam and Nepal. However, in the case of China, Hu (2007) conducted an examination of China’s foreign language policies by utilizing publications, official documents, and interviews with officials. The study revealed that China’s Ministry of Education mandates English as a compulsory subject starting from grade 3, with the policies being shaped by social, educational, economic, political, and linguistic factors. These findings served as a guiding framework for this research question. Furthermore, Hou (2012) highlighted the existence of an English curriculum resource disparity between urban and rural primary schools over the years due to the interpretation of policies related to PEE by the Chinese Ministry of Education. In this regard, emphasis should be placed on teacher development and training as the core aspect of a balanced education resource strategy. These questions and suggestions reflect both the policy implementation situation 10 years ago and resource allocation issues, providing a foundation for designing this research questionnaire.

PEE places greater emphasis on the education of primary English teachers (Emery, 2012; Kabilan and Veratharaju, 2013; Enever, 2014; Sah, 2022). Emery’s (2012) study investigated the qualifications, teaching experience, training, and career development of primary English teachers through an electronic survey. The findings indicated that a class size of fewer than 35 students is insufficient for most teachers and highlighted the need for degrees, specific training, and professional development. However, addressing the developmental needs of teachers in Malaysia’s primary schools, Kabilan and Veratharaju (2013) discovered that English language teachers aspire to meet the requirements of schools, students’ needs as well as their own teaching practices. These studies have effectively illustrated the challenges faced by English language teachers. More recently though, Sah (2022) emphasized Nepal’s requirements for primary school teachers who are considered generalist educators needing to obtain a license through TSC with eight essential competencies including content knowledge, pedagogy skills, knowledge about children, classroom management skills, effective communication and collaboration abilities, professional development opportunities, adherence to professional conduct standards, and proficiency in information technology along with awareness towards diversity among learners. This highlights how in-service teacher training programs and pre-service university courses may not adequately prepare educators for linguistic, cultural diversity within classrooms due to educational policies in Nepal focusing primarily on pedagogical techniques, English language proficiency levels, instructional methodologies, and material design.

Indeed, there are escalating challenges in primary English education (PEE). For instance, Sulistiyo et al. (2020) conducted interviews with six teachers from an Indonesian primary school and identified various issues in primary education. These include the absence of official curriculum guidelines, inadequate learning resources and facilities, as well as concerns regarding the quality of English teachers. Do and Lê (2012) reported that a significant majority of primary English teachers in Vietnam (65.4%) lack sufficient training and proficiency in English language skills and pedagogic techniques. The implementation of policies at the commencement of grade 3 encounters obstacles such as teacher shortages, insufficient professional development opportunities for educators, as well as limitations in teaching resources, methodologies, and materials (Nguyen, 2011). In China’s context, there is a pressing need to enhance the professional competence, classroom instruction effectiveness, and management abilities of primary school English teachers. Additionally, teaching conditions remain underdeveloped in impoverished counties within Yunnan Province (Cai et al., 2012). Moreover, Yang and Xiong (2009) conducted a comprehensive survey comprising questionnaires, observations, and interviews to assess the professional competence and classroom effectiveness of English teachers in 39 primary schools located in the urban area of Wuhan. The study identified several key issues: Firstly, learners have limited exposure to English due to the relatively short duration of primary school English classes which last only 240 h on average with an overcrowded class size of approximately 50 students. Consequently, learners’ proficiency levels in English exhibit significant disparities. This discrepancy can be attributed primarily to the fact that many English teachers are not specialized in teaching English but rather possess backgrounds as general educators, preschool instructors or art teachers who lack sufficient proficiency in spoken English including pronunciation and intonation skills. As a result, they encounter difficulties expressing themselves fluently during classroom instruction while struggling to design instructional strategies aligned with the cognitive patterns characteristic of primary school students. Furthermore, these dedicated educators face heavy workloads with weekly teaching hours ranging from 18–24 for most primary school English teachers; additionally, around 60% of them are responsible for instructing multiple grades simultaneously. Some even find themselves burdened with additional responsibilities such as teaching other subjects or serving as class supervisors.

The research pertaining to professional English education (PEE) often centers on addressing the challenges associated with this field. Emery (2012) put forward several recommendations for enhancing learning and teaching in PEE, including the recruitment and training of highly qualified teachers, implementation of a rotational teaching system, organization of diverse workshops for teacher professional development, provision of more opportunities for career advancement, and meeting the needs of teachers. Additionally, Wei (2013) emphasized the significance of strengthening research on the professional growth of English teachers in rural primary schools to enable education departments at all levels to accurately comprehend their current situation regarding professional development. This will facilitate proactive measures being taken to enhance their professional competence and effectively address difficulties encountered during the implementation of new English curriculum guidelines. Ultimately, these efforts aim to improve both the effectiveness of English curriculum implementation and the quality of English instruction in rural schools.

PEE has become indispensable in educational research due to its inherent value. For instance, Qi (2016) examined the significance of English classes by comparing the frequency of weekly lessons for English, Chinese, and mathematics-the three core subjects in primary education-as well as exploring urban–rural disparities among families, schools, and children. The findings reveal that although English is ranked lower than Chinese and mathematics, parents hold high expectations for English learning due to the belief that “the earlier one starts learning English, the better.” Furthermore, an examination-oriented approach to teaching English encourages students to prioritize their language acquisition.

Moreover, PEE not only offers solutions to problems but also encompasses a broader scope. For instance, Valente and Xerri (2022) authored the book *Innovative Practices in Early English Language Education*, which comprehensively addresses various aspects of early English language education for children aged 3 to 12. The book delves into topics such as teaching methods and theories, classroom practice, curriculum development, child-centered assessment, pre- and in-service teacher education context, among others. While the researchers extensively discuss educational content like developing critical thinking skills, providing teacher support, fostering learner-centeredness, offering diverse materials and approaches as well as incorporating children’s perspectives; they overlooked the aspect of school support and further empirical research that warrants exploration.

Due to the utilization of diverse methodologies and procedures in the aforementioned studies, it is intriguing that a majority of the research has primarily concentrated on investigating educational policies, English teacher development, as well as educational issues and corresponding solutions. Additionally, there have been investigations conducted regarding the significance of PEE and early language education. Nevertheless, only a limited number of studies pertaining to PEE in the Hunan Province and the sustainable development of PEE alongside the CIPP model can be identified.

### 1.1 Primary English education in the Hunan Province

Li and Wang (2009) conducted a survey on the current status of primary school English teachers in nine urban areas and some rural primary schools in the Hunan Province, including Changsha, Xiangtan, Hengyang, Changde, and Zhangjiajie, from 2002 to 2008. Among the 2,933 primary schools surveyed, it was found that there should be a total of 9,076 primary school English teachers according to the Ministry of Education’s requirements. However, there was an actual shortage of 6,403 teachers resulting in a vacancy rate of 71%. Additionally, out of the total number of surveyed teachers (2,673), only 134 possessed a bachelor’s degree or higher qualification which accounted for merely 5.01%. Furthermore, there were also only 883 undergraduate, junior college, and vocational school teachers with English professional backgrounds accounting for approximately one-third (33%) among all surveyed teachers as well as representing just about one-tenth (9.7%) when compared to the required number set by the Ministry of Education for primary school English teachers. Although their research provided insights into the basic situation regarding primary English teaching staff which can serve as foundational knowledge for current studies; however their focus did not specifically address Primary English Education. Tang (2011) highlighted the low level of development among English teachers in rural primary schools in the Hunan Province. In order to promote rapid economic and social development in Hunan and accelerate the progress of rural compulsory education, it is crucial to adopt various teacher training modes for English teachers in rural primary schools. This emphasizes the significance of teacher development while addressing issues and proposing solutions within this domain. Fan (2010) conducted a survey on the fundamental situation of the primary school English teacher team in Changsha, Hunan Province, encompassing the recognition of frontline primary school English teachers, the significance of the school to the English subject, and the career prospects of primary school English teachers. He acknowledged the triumph of PEE reform in Furong District, Changsha City by summarizing that its progress was due to superior material and teaching conditions, advanced teaching equipment, and an early start to classes. The values held by schools and teachers exhibited a high level of awareness towards professional growth which provides a valuable reference for research on PEE.

In recent years, there have been a limited number of studies conducted by Chinese scholars on the current state of English education in primary schools. Several investigations have focused on the status quo of English teacher education in Qianxinan Prefecture, Heze City, Chongqing City, Chifeng City, and rural areas of China (Chen, 2020; Fu, 2020; Sai, 2020; Cai, 2021; Sun and Zhang, 2023). These studies commonly assert that rural primary schools in China face challenges such as inadequate English teachers and insufficient teaching resources resulting in a relatively underdeveloped level of English education. However, few scholars have explored the present situation of English education in primary schools specifically within the Hunan Province.

Although the majority of research on primary English education (PEE) in the Hunan Province has primarily focused on teaching issues, there have been studies conducted in various areas. Li and Wang (2009) as well as Tang (2011) specifically examined the teachers involved in PEE in Hunan, highlighting concerns such as their limited professional backgrounds and teacher shortages. While Fan (2010) argued that the pilot program for primary school English teaching was highly successful, it is valuable to learn from the experiences of teachers, the teaching environment, and curriculum arrangements. In recent years, few studies have explored the quality of PEE at a micro level within both rural and urban primary schools.

### 1.2 An overview of CIPP model

In 1966, the renowned American educational evaluator D.L. Stufflebeam introduced the goal-oriented CIPP evaluation model and emphasized that the purpose of evaluation was to enhance and gather information about the entire process and outcomes of educational program implementation (Lu, 2009). This model comprises four stages: context evaluation, input evaluation, process evaluation, and product evaluation (Jin, 2017). The initial step involves background assessment to evaluate the alignment between course implementation background and users’ needs while providing a reference for leaders’ decision-making based on this alignment. The second step is input evaluation which builds upon background assessment to evaluate the anticipated effectiveness of course implementation and offer decision-making support for leaders. The third step entails process evaluation during implementation to aid leaders in understanding plan progress and making improvements. Lastly, it determines whether the course plan has achieved expected results (Xu and Liao, 2022). Currently, the CIPP model is primarily utilized for ideological and political evaluations of courses (Pan and Xue, 2023), clinical nursing teaching quality evaluations (Lei et al., 2023), teaching reflection evaluations (Yang and Kang, 2023), construction of educational evaluation indicators (Chen and Shao, 2023), etc. However, research on English education based on the CIPP model predominantly focuses on college-level English (Yin, 2019; Wu, 2022) as well as middle school English evaluations (Li et al., 2011), with most studies exploring the theoretical aspects of the CIPP model. Regarding primary English evaluations based on the CIPP model specifically, Nan and Song (2014) analyzed course purposes, designs, processes, and outcomes, focusing also on theory, and proposed a multi-dimensional approach to assess course reform effects. Li (2002) underscored the significance of adopting a comprehensive and diverse approach to curriculum evaluation. She posited that this multidimensional assessment should encompass language proficiency, educational environment, interpersonal relationships, emotional well-being, individual learner characteristics, and learning strategies. Her research sheds light on the importance of involving students in the evaluation process not only for fostering motivation but also for assessing their abilities as an educational objective. Moreover, it is noteworthy that primary English education differs from PEE as it encompasses a broader scope and focuses on the entire teaching process along with associated factors. Therefore, employing the CIPP model for evaluating primary English education is deemed more pragmatic.

### 1.3 Development of sustainability in primary education

An essential concern lies in the definition of sustainable development and sustainable development education. Since the 19th century, research on sustainable development has revolved around ecological issues, emphasizing the concept of equitable development and a future-oriented approach. Since the late 20th century, the scope of “sustainable development” has broadened to encompass fields such as economy, politics, and culture, advocating for enhancing the quality of future societal progress (Han et al., 2002). The characteristics of sustainable development can be summarized as follows: firstly, achieving equitable development for all and narrowing resource allocation disparities among peers; secondly, continuously strengthening humanity’s capacity to advance towards a better world.

The process of sustainable development education involves imparting knowledge on sustainable development to students, fostering their awareness and abilities in this field (Shi, 2003), and enabling them to develop a cognitive understanding and practical skills for achieving sustainable development through theoretical knowledge (Tian and Zhao, 2009). This empowers them to assume the responsibility of shaping the future (Wang and Li, 2011). Furthermore, as defined by Alan Gilpin in the *Dictionary of Environment and Sustainable Development* in 1996, sustainable development encompasses both living and non-living resources with a focus on conservation as well as weighing the pros and cons of alternative actions for future generations. Gilpin also emphasizes the pedagogy of sustainability which prompts individuals to address unresolved challenges or “traps” while encouraging critical thinking among students and teachers. Therefore, this study defines sustainable development education as the utilization of diverse resources by educators in educational activities to provide professional knowledge, guidance on activities and abilities to learners with the involvement of multiple stakeholders. It focuses on addressing challenges encountered in students’ learning, promoting educational equity, bridging resource gaps, and enhancing learners’ sustainable development in terms of knowledge, abilities, values, and behaviors through reflection and improvement. The characteristics of sustainable development education lie in its practicality and universality. In terms of practicality, research has indicated that sustainable development education should commence with real-world issues and achieve sustainability by resolving them (Wang and Zhong, 2004). Universality is reflected in the need for increased public participation such as government provision of adequate materials and financial support for sustainable development (Tian and Yang, 2010). This study’s investigation into sustainable development begins by examining the current state of English education practices in primary schools while considering educational participants such as schools themselves, curriculum designations, and teachers and students involved,. Through an examination involving both students and teachers we gain insight into the shortcomings present within English education across various primary schools within the Hunan Province while also calling for problem-solving measures to promote PEE’s sustainable development.

The development aspect of sustainable development education should encompass educational methodologies, content, evaluation, and other related factors. For instance, in the “Introduction to Sustainable Development Education at Beijing 101 Middle School,” the objectives, content, evaluation criteria, support measures and other relevant aspects of sustainable development education are clearly defined. This implies that education must undertake curriculum design, school activities and external exchanges for promoting sustainable development while organizing targeted student visits abroad to exchange ideas which have yielded positive results in teaching sustainability. Existing research on sustainable development education in primary schools mainly focuses on exploring this concept within a specific discipline by integrating it into educational goals, content and teaching methods. In the implementation process, there is a strong emphasis on fostering student participation and exploration. Xu (2013) immersed himself in research and social practice within primary school science education activities to integrate the concept of sustainable development education. The optimization of the teaching process, diversification of teaching methods, and enhancement of teacher quality are advocated by Zhang (2004) to foster sustainable development in middle school geography education. In her study on the sustainable development of primary school English classes, Cao (2019) explored students’ English learning abilities based on multiple learning theories and proposed that teachers should prioritize imparting effective learning strategies to unleash students’ initiative and subjectivity. Additionally, studies have examined the sustainable development of primary school English teachers’ professional growth at an individual level through questionnaire surveys regarding their educational backgrounds. These studies highlight the importance of improving cooperation awareness, practical ability among teachers, teaching proficiency, professional judgment ability, as well as dynamically promoting teacher’s professional development (Mo, 2009). Scholar also advocates for schools and educational institutions to provide continuous professional development and training opportunities for teachers, as well as to research and innovate educational assessment methods, aiming to further enhance the quality of primary English education and student outcomes in sustainable development (Li, 2023). The findings of these studies serve as a fundamental framework for advancing the sustainable development of English education in primary schools in the Hunan Province, employing sustainable development concepts and strategies.

Currently, research on sustainable development education is progressively deepening; permeating various disciplines, and holds significant theoretical and practical implications for promoting the sustainability of education. However, there is a lack of sufficient research specifically focused on the sustainable development of English education. Moreover, the scarcity of research on the sustainable development of primary school English education in the Hunan Province necessitates further enrichment. In terms of theoretical exploration, the field of primary English education’s sustainable development theory is still in its infancy. A search conducted on CNKI revealed less than 10 relevant literature pieces concerning the sustainable development of primary English education; furthermore, no comprehensive theoretical framework or project practice was found to exist. The existing research primarily revolves around educational content and theory at the primary school level while lacking pertinent practical investigations.

The questionnaire content for students and teachers needs to be carefully considered, taking into account the principles of Marxist theory on all-round human development as well as student development theory. According to the Marxist perspective, human quality encompasses physiological, psychological, social, and ability aspects (Shi, 2002). Primary school English education should prioritize fostering comprehensive development in students by nurturing their cognition, abilities, and behaviors while promoting harmonious and unrestricted individual growth. Additionally, in the 1920s, American colleges and universities embraced the student development theory to guide employment activities with a shared belief that cultivating scientists is a joint responsibility among students themselves, school administrators, and the government. This theory is categorized into four types: personal and environmental theory, social public psychology theory, cognitive and value theory, and integration theory (Renn, 2008). Among them, the individual and environment theory emphasizes the correlation between students and their surroundings. Astin has proposed an IEO model that accentuates both the environment and student involvement by focusing on the survey sample’s personal background, learning motivation, educational and social interaction environment as well as their learning outcomes and personal growth after being nurtured in a specific setting (Renn, 2008). According to student development theory, students should be provided with challenges along with support including personnel assistance and resources (Renn, 2008). Throughout students’ learning process, school support, course arrangements, teacher-student participation are all integrated aspects that should be included in educational surveys.

Integrating the aforementioned theory of sustainable development with Marx’s comprehensive human development theory and student development theory establishes a theoretical framework for the sustainable development of primary school students. Guided by the objective of sustainability, primary school students’ education should take into account the impact of their environment, curriculum, and teachers on their English learning process. They should fully harness their own initiative, foster an interest in learning, impart relevant knowledge, cultivate their abilities, and develop behavioral habits to facilitate the holistic and enduring advancement of their English proficiency.

In summary, there is currently a limited body of research on the sustainable development of English education in primary schools, particularly within the Hunan Province. According to Marx’s theory of comprehensive human development and student theory, as well as sustainable development theory, the sustainable development of education necessitates an integrated investigation into the environment, curriculum, teachers’ qualifications and students’ learning. CIPP places emphasis on quality assessment throughout the educational process. This paper constructs a model for the development of primary school English education within the theoretical framework of CIPP and applies this model in practice to evaluate the current state of primary school English education in the Hunan Province. Accordingly, based on the framework of the CIPP model, the following questions will be proposed:

Question 1: What is the existing condition of PEE in the Hunan Province according to the CIPP model?

Question 2: What are the deficiencies and causes for the sustainable development of PEE in the Hunan Province?

Question 3: What improvement suggestions can be put forward to address the problems in PEE?

## 2 Methodology

The present study aims to conduct a questionnaire survey on primary school English teachers and students in the WeChat app. Additionally, based on the CIPP evaluation model, this study will analyze the current situation, shortcomings, and underlying factors of English education in the Hunan Province.

### 2.1 Participants

This study randomly selected participants in primary schools from 13 regions in the Hunan Province for investigation. The participants were 42 primary school English teachers and 404 primary school students from different grades in 90 urban and rural primary schools in 13 regions in the Hunan Province, which is in the south part of China. Among the participants, 45.24% (*n* = 19) of the surveyed teachers came from urban primary schools, and 54.76% (*n* = 23) came from rural primary schools. The 13 areas involved in the investigation were Changsha, Yongzhou, Hengyang, Shaoyang, Yiyang, Yueyang, Zhuzhou, Loudi, Huaihua, Changde, Chenzhou, Zhangjiajie, Xiangxi Tujia, and Miao Autonomous Region in the Hunan Province. The 90 schools involved in the investigation included Yanshan Primary School, Datong Primary School, Wangling Central Primary School, Taiping Primary School, Yunji Central Primary School, and other urban and rural primary schools. Table 1 reports the gender, age, educational background, English educational background, length of teaching experience, technical post, and workplace of the participating teachers.^1^

**Table 1:** Teachers’ demographic characteristics (*n* = 42).

Demographic characteristics		Number	Percentage
Gender	Male	9	21.43%
	Female	33	78.57%
Age	Under 40 years	34	80.95%
	Over age 40	8	19.05%
Education	Other	1	2.38%
	Junior college	3	7.14%
	Bachelor	37	88.1%
	Master	1	2.38%
	Doctorate	0	0%
English major education	Other	5	11.9%
	Less than technical secondary school education	1	2.38%
	Vocational school education in English major	0	0%
	Junior college education in English major	4	9.52%
	Bachelor’s degree in English major	31	73.81%
	Master’s degree or above in English major	1	2.38%
Length of teaching experience	0–3 years	13	30.95%
	4–6 years	9	21.43%
	7–9 years	9	21.43%
	Over 10 years	11	26.19%
Technical post	Third level	7	16.67%
	Second level	13	30.95%
	First level	17	40.48%
	Senior	5	11.9%
Workplace	Rural area	23	54.76%
	City	19	45.24%

For teacher participants, in order to ensure a more representative sample, the study considered teachers of diverse age groups, genders, and job titles who are actively engaged in frontline English teaching at primary schools. Additionally, they should demonstrate willingness to actively cooperate with the investigation and have long-term employment in their respective schools. Teachers who were absent or primarily involved in administrative work during the data collection period will be excluded from the study. Student participants encompass students from all grades currently receiving English language education who willingly participate in the investigation (See Table 2).^2^

**Table 2:** Students’ demographic characteristics (*n* = 404).

Demographic characteristics		Number	Percentage
Grade	1	5	1.24%
	2	7	1.73%
	3	33	8.17%
	4	231	57.18%
	5	22	5.45%
	6	106	26.24%

### 2.2 Procedure

The present study was conducted in three stages. The first stage spanned from July 2019 to December 2020, during which a comprehensive research plan was formulated, specialized research activities were carried out, and data collection took place. The second stage extended from January 2021 to July 2022, with the primary focus being on gathering education evaluation data to gain insights into the current state of educational quality. Additionally, the collected data underwent analysis across four dimensions—context evaluation, input evaluation, process evaluation, and product evaluation—aiming to explore key and challenging issues outlined in the research plan. Lastly, the third stage encompassed August 2022 to May 2023 wherein an assessment of results based on the collected data was conducted followed by writing conclusive findings.

### 2.3 Instrument

In determining the dimensions of the questionnaire, this study conducted a survey from the perspective of sustainable development practice, considering educational objectives, educational content, teaching methods, and teaching evaluation based on the four levels of CIPP theory. Additionally, it incorporated the general teaching process and comprehensive development theory. Given the common characteristics of sustainable development, active involvement of multiple subject resources such as schools, curriculum, teachers, and students is essential. When designing the questionnaire items for primary school education activities in terms of educational goals, content, methods and participation of multi-subject resources; relevant research on sustainable development education and primary school sustainable development education was consulted.

**Figure 1:**
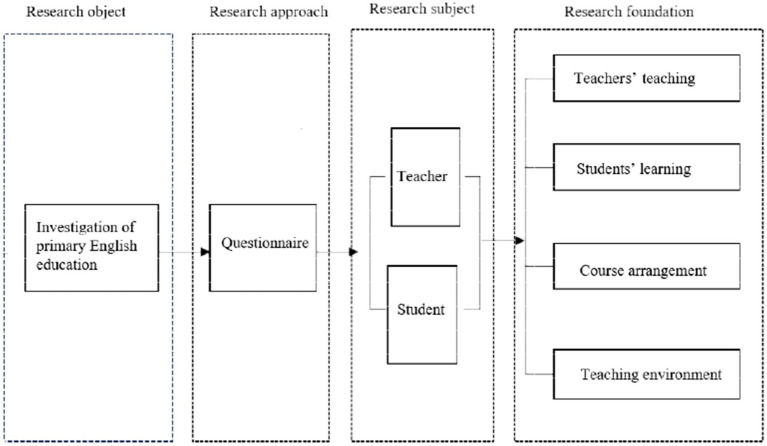
The research content of PEE.

The questionnaire survey on the current state of PEE comprised questionnaires targeting both teachers and students. The questionnaire content covered various aspects, including teacher profiles, student demographics, curriculum details, and environmental factors. Figure 1 illustrates the research scope of PEE.

The teacher questionnaire included a total of 38 questions, with 25 single-choice questions (TQ3–TQ27), 5 multiple-choice questions (TQ28–TQ32), and 8 broad open questions (TQ1, TQ2, TQ33–TQ38). Among them, the first and second questions were about the investigation of the schools’ geographical location. The survey questions for basic information about teachers were TQ3, TQ4, TQ5, TQ6, TQ7, TQ8, and TQ9. The student questionnaire consisted of 17 questions, including 13 single-choice questions (SQ3–SQ15), 2 multiple-choice questions (SQ16–SQ17), and 2 broad open questions (SQ1, SQ2). The first and second questions were about investigating the geographical location of the school, while SQ3 was about investigating the basic information of students.

### 2.4 Data collection and analysis

The Hunan Province is located in southern China, with a total area of 211,800 square kilometers, 13 prefecture-level cities, and one autonomous prefecture under its jurisdiction. To make the collected data representative, the 13 regions selected came from different prefecture-level cities or prefectures in the Hunan Province. Therefore, the primary school students and teachers came from both economically developed urban areas and economically underdeveloped rural areas. The survey arranged for 13 people who were asked to contact the teachers and students of the surveyed schools through questionnaire distribution and on-site visits, inviting them to complete the questionnaire. Because of the wide range of data collected, there were difficulties with the survey; the data collection and analysis took a total of 2 years, and 90 schools in 13 regions in the Hunan Province were surveyed. Relevant information was collected from 42 teachers and 404 primary school students.

The teachers and students were invited to use Questionnaire Star (software for conducting questionnaire surveys on mobile phones) to complete the electronic questionnaire, and they were requested to choose or fill in the survey content according to the actual situation throughout the year. During the data collection process, for elementary school students who had difficulty understanding questions, especially those in lower grades, the investigator explained to them and assisted them in completing the questionnaire with the help of the class teacher. For teachers or students who had operational difficulties completing the questionnaire, the investigator provided assistance in operation and question understanding.

To ensure the quality of the data received, the study group members checked all the questionnaires to ensure that the data is complete, and the questionnaires collected before the deadline, the questionnaires meet the requirements of the purpose, and checked the integrity of the data derived from the Questionnaire Star; the group members also try to exclude uniform answer questionnaires and questionnaires with inconsistent answers. To analyze the question—the existing condition in PEE in the Hunan Province—the total frequencies of various educational situations were tallied for each teacher’s and students’ group. The features representing problems were recorded qualitatively, which helped us analyze the educational situation.

## 3 Results and discussions

**Figure 2:**
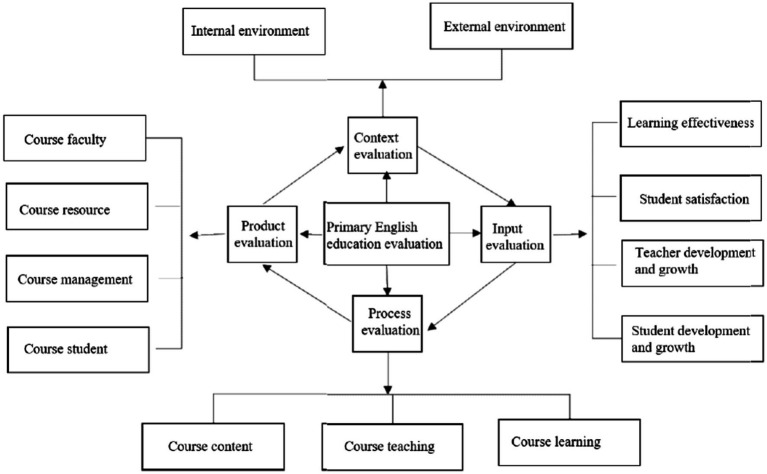
The assessment content for PEE.

With a focus on addressing the challenges facing primary school English education in the Hunan Province, this study has developed a CIPP model for assessing educational quality based on an extensive review and analysis of relevant literature. Figure 2 demonstrates the assessment content for PEE.

According to the CIPP evaluation theory proposed by American scholar Stufflebeam, the evaluation process can be categorized into four components: contextual evaluation, input evaluation, process evaluation, and product evaluation. This paper, based on the CIPP framework and the foreign language curriculum teaching evaluation model (Yin, 2019), constructs assessment indicators focusing on teacher instruction, student learning outcomes, curriculum design effectiveness, learning environment creation, and school facilities quality. Additionally, a questionnaire is designed to investigate the progress of primary school English education for both teachers and students. Table 3 classifies the coding scheme for investigation episodes from a CIPP angle.^3^

**Table 3:** Coding scheme for investigation episodes^3^.

Categorization	Conceptualization	Description
Context evaluation Internal environment External environment	Student learning experience Students’ English foundation Students’ interest and motivation Policy positioning School support Curriculum positioning	SQ4: Have you ever studied English in kindergarten/preschool? SQ5: Have you ever/currently participated in English tutoring at a training institution? TQ21: How about your students’ English pronunciation? TQ27: What is the interest and motivation of primary school students in learning English? TQ11: How much importance does your school attach to English? TQ12: Do you think primary schools should offer English classes? TQ36: What do you think is the position of English courses in your school compared to other subjects?
Input evaluation Course faculty Course resource Course management Course student	Faculty level Faculty size Faculty structure Faculty training Faculty work scope Teaching facilities Course materials Course arrangement Learning input	TQ20: How about your English pronunciation? TQ10: What is the shortage of English teachers in your school? TQ3: Is your gender male or female? TQ4: What is your age range? TQ5: What is your highest level of education? TQ6: What is your major? TQ7: Which range does your teaching experience belong to? TQ8: What category is your professional title? TQ9: Is the primary school where you work located in rural or urban areas? TQ32: What continuing education have you participated in? TQ13: Do you work as a class teacher? TQ29: What teaching equipment do you use in the classroom? TQ17: What version of the English textbook are you currently using? TQ28: What textbooks have you used before? TQ14: How many grades of English classes do you teach? TQ15: How many English classes do you have per week? TQ16: Do you teach any other subjects besides English? SQ7: How many English classes do you have per week this semester? SQ14: How many minutes do you spend on English learning every day?
Process evaluation Course content Course teaching Course learning	Course difficulty Course feedback Teaching methods Teaching means Student participation Learning method	TQ33: What difficulties have you encountered in English teaching? TQ34: What do you think should be paid attention to during the teaching process? TQ18: What language do you use for teaching in the classroom? TQ31: What teaching methods do you use in your teaching? TQ30: What teaching aids do you use in the classroom? SQ11: Do you communicate in English with teachers and classmates in English class? SQ16: What are you commonly used learning methods?
Product evaluation Learning effectiveness Student satisfaction Teacher development and reflection Student development and reflection	Ability goal Knowledge goal Emotional goal Course implementation Teaching reflection Learning reflection	SQ10: Can you understand the content taught by the teacher in English Class? TQ25: How are students learning English? TQ26: How effective is learning English for primary school students? SQ8: Do you enjoy learning English? SQ9: Are you willing to take English classes? SQ12: Are you willing to communicate with teachers and classmates in English class? SQ15: Do you think learning English has increased the learning burden? TQ19: Which teaching method do you think is more effective in class? TQ22: Do you think students’ pronunciation is greatly influenced by teachers? TQ23: How do you evaluate your teaching? TQ24: Do you think you are capable of teaching English? TQ35: What advantages and disadvantages do you think primary school students have in learning English? TQ37: What do you think are the main problems in primary school English education? TQ38: Do you have any suggestions for primary school English education? SQ13: Are you good at learning English? SQ17: What are your difficulties in learning English?

TQ: teachers’ question in questionnaire; SQ: students’ question in questionnaire.

Drawing on the CIPP evaluation model and teaching evaluation indicators proposed by Yin (2019), our research group extensively discussed and validated the PEE evaluation indicators and questionnaire items, resulting in the final version of the questionnaire. To ensure its feasibility, a pre-test was conducted with 10 students, and feedback was used to make further modifications.

Based on the successful implementation of the pre-test questionnaire, a questionnaire survey was conducted in 13 regions of the Hunan Province. The collected questionnaires were carefully screened and analyzed, resulting in the selection of 42 valid teacher questionnaires and 404 valid student questionnaires. This study aimed to assess the validity of the questionnaire results. Validity analysis was conducted on the single-choice questions in the teacher questionnaire, with KMO values ranging from 0.665 to 0.965, indicating that the data information is suitable for extraction. The multiple-choice question *χ*^2^ = 23.949, *p* = 0.000 in the teacher questionnaire, with a *p* value of less than 0.05, indicates a significant difference in the proportion of various options. The validity analysis of the single-choice questions in the student questionnaire showed that the KMO values were 0.861–0.993, and the data were all greater than 0.8, indicating that the validity is very good and very suitable for extracting information. The multiple-choice question *χ*^2^ in the student questionnaire is 8.963, *p* = 0.003, and its *p* value is less than 0.05, indicating a significant difference in the proportion of various options.

Through the analysis of the questionnaire, this paper explores the current state of PEE in the Hunan Province, evaluates its present status based on the CIPP model, and investigates the identified issues along with corresponding countermeasures for their resolution.

### 3.1 Research question one addresses the current situation of PEE in the Hunan Province

According to the CIPP educational evaluation theory proposed by D.L. Stufflebeam in 1966, a contextual evaluation serves as the backdrop for course implementation, encompassing a comprehensive understanding of the policy background, practical needs background, and environmental background. It further delves into the conditions and resources required for successful implementation (Xu and Liao, 2022). The context includes both external and internal environments. The external environment is influenced by national policies, public opinion, as well as schools’ support and attitude towards education. On the other hand, the internal environment pertains to students themselves and forms their foundation (Chen and Shao, 2023). Drawing from student development theory emphasized by Astin which highlights environmental factors and student participation; this study focuses on personal backgrounds, learning motivation, educational environment, and social interaction environment (Renn, 2008). Therefore, in this study’s assessment of context encompasses both internal aspects such as students’ learning experience English proficiency level along with their interest and motivation; as well as external elements like policy positioning school support curriculum alignment primarily examining clarity in policies environments curriculum positioning.

After analyzing the internal environment, it was discovered that primary school students demonstrate variations in their foundational learning, which will impact the sustainable development of PEE. Learning interest plays a crucial role in maintaining students’ sustainable progress, and a majority of the surveyed students have expressed an inclination towards English language acquisition. Out of the 404 primary school students surveyed, 25.74% (*n* = 104) had already received English instruction during their time in kindergarten or preschool, while 47.28% (*n* = 191) were either currently enrolled or had previously participated in English training programs. According to survey data, 69.05% (*n* = 29) believed that student pronunciation was deemed as standard; however, there were also concerns raised by 28.57% (*n* = 12), who felt that student pronunciation fell short of being considered standard. Furthermore, teachers perceived a genuine interest among students towards learning English with percentages indicating high levels of interest at 19.05% (*n* = 8), relatively high levels at 45.24% (*n* = 19), and general interest at 33.33% (*n* = 14). In this context, 55.2% (*n* = 223) of the students expressed an active desire to learn English while 37.38% (*n* = 151) did not reject the idea.

In terms of the external environment, currently, English classes are highly valued in most primary schools. According to the survey results, 88.09% (*n* = 37) of respondents stated that their school places importance on English classes, with 28.57% (*n* = 12) indicating a significant emphasis on them. Furthermore, an overwhelming majority of teachers (97.62%, *n* = 41) agreed that primary schools should offer English classes. The opinions among teachers varied; however, the majority regarded English classes as a core subject alongside math and Chinese classes and acknowledged their relative significance. Nonetheless, a minority of teachers expressed concerns regarding the lack of seriousness towards English education.

The process of input evaluation involves investing in the teaching process and is a crucial step in establishing the foundation for curriculum development (Xu and Liao, 2022). Input evaluation focuses on assessing resource allocation, such as institutional settings, rules and regulations, human resources, funding investment, implementation investment, and more (Xu and Liao, 2022). According to student development theory (Renn, 2008), students should receive support in terms of personnel and resources. Additionally, sustainable development theory emphasizes the importance of providing material resources and support. All aspects related to resources fall within the scope of input evaluation: course faculty (faculty level, size, structure, training programs), course resources (teaching facilities and materials), course management (arrangement), and course students’ learning inputs. This evaluation aims to determine whether teaching resources meet practical needs effectively while considering targeted outcomes and students’ time investment in learning.

Regarding the course faculty, teachers play a pivotal role in ensuring the sustainable development of primary school English education. While there may be a shortage in the number of English teachers, there has been an improvement in their quality and increased emphasis on training. The structure and responsibilities assigned to teachers are relatively reasonable and align with the overall goals of current education. Out of the 42 surveyed teachers, 38 possessed a bachelor’s degree or higher, accounting for a total proportion of 90.48%. Additionally, 32 had obtained a bachelor’s degree or higher in English studies, representing 76.19% of the total sample size. Teachers with backgrounds in English studies accounted for 85.71% (*n* = 36), regardless of whether they held college degrees, undergraduate degrees, or master’s degrees. Regarding the proficiency level of teachers, a significant majority of 92.86% (*n* = 39) out of the surveyed group of 42 teachers expressed confidence in their own pronunciation, affirming that it adhered to standard norms. In terms of faculty size, among the same group of 42 teachers, approximately 76.19% (*n* = 32) reported a shortage of less than three educators within their respective schools. Notably, there existed a considerable disparity in teacher numbers across the ten schools examined, with all surpassing four instructors each. With regards to faculty training, an overwhelming emphasis was placed on continuous education and learning by these educators; as evidenced by an impressive participation rate of 90.48% (*n* = 38) in teaching training programs—which may serve as a crucial factor contributing to the unified enhancement of teaching methodologies employed by them. Furthermore, within this context, it is worth noting that educational promotion activities were undertaken by approximately 38.1% (*n* = 16) of these teachers. In addition, 2.38% (*n* = 1) pursued further education abroad, while 30.95% (*n* = 13) of teachers engaged in various forms of training, including educational technology training and ideological and political training, among others. Among the faculty members, there were 26 teachers (61.9%) who solely taught English classes without taking on class teacher responsibilities. Regarding the composition of the faculty, the male-to-female ratio was 1:3 and approximately 80.95% (*n* = 34) of teachers were under the age of 40. Furthermore, a total of 90.48% (*n* = 38) possessed a bachelor’s degree or higher qualification overall; specifically for English qualifications alone, this percentage stood at 76.19% (*n* = 32). Moreover, around 69.05% (*n* = 29) of teachers had more than three years’ work experience and 52.38% (*n* = 22) held a first-level teacher or higher professional title. Furthermore, 45.24% (*n* = 19) of teachers worked in urban areas whereas 54.76% (*n* = 23) worked in rural areas.

**Figure 3:**
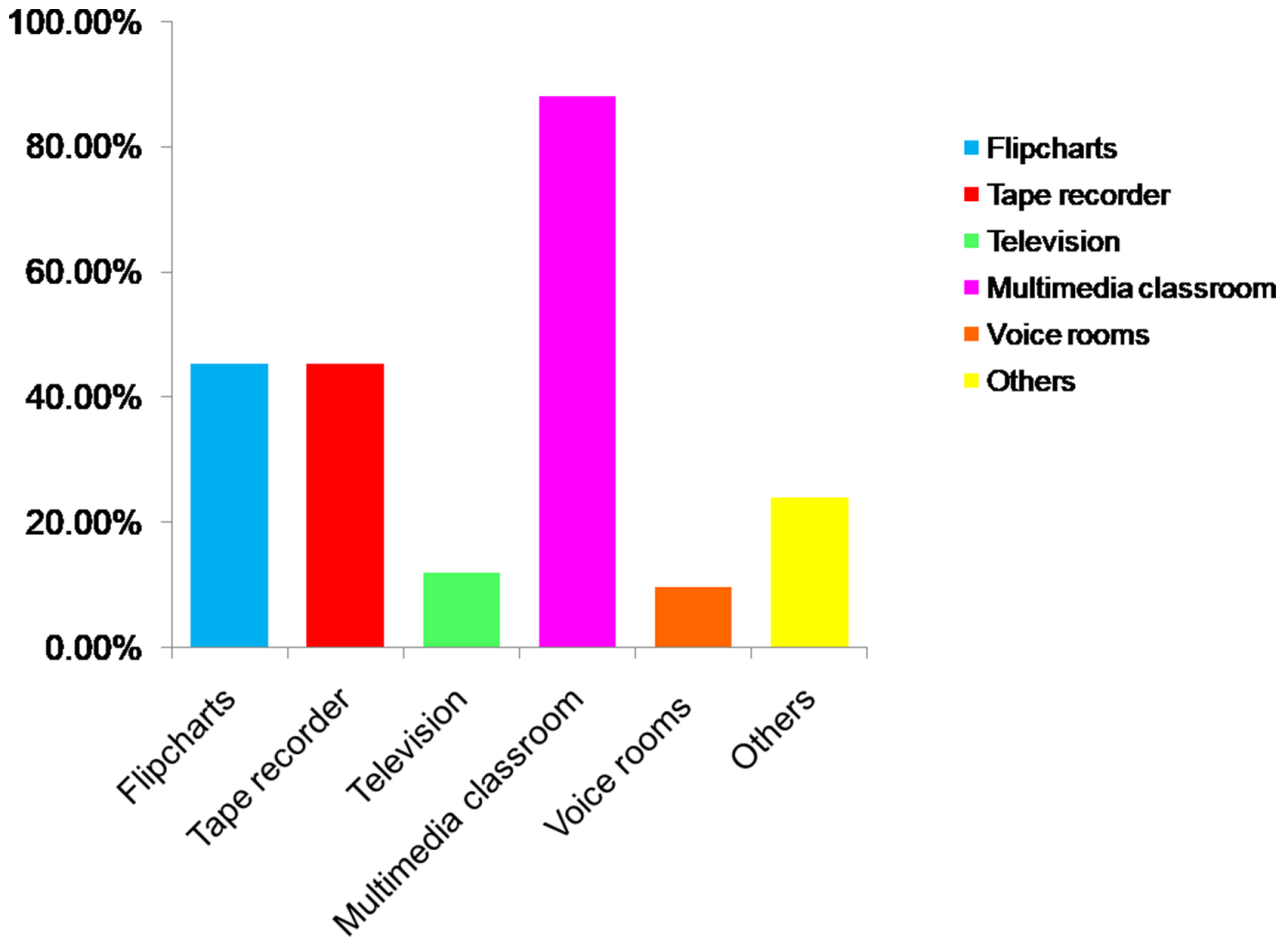
The equipment being used by teachers in English classes.

In the context of sustainable development, the utilization of teaching equipment and textbooks by educators also facilitates students’ English language proficiency. This investigation revealed disparities in terms of teaching resources across different educational institutions, with a majority lacking modern amenities such as voice rooms. The instructional materials employed by teachers predominantly consist of nationally-published textbooks from People’s Education Press and local publications from Hunan Education Press. Regarding course resources, teachers employ a diverse range of teaching equipment, with multimedia classrooms being the most prevalent at 88.1% (*n* = 37) usage rate among all available options. However, due to financial constraints faced by most schools, only a mere 9.52% (*n* = 4) of surveyed teachers had access to voice rooms. Figure 3 reports the equipment being used by teachers in English classes.

The textbooks published by People’s Education Press and Hunan Education Press have become the primary teaching materials for teachers, while PPT courseware and card teaching serve as the main teaching aids in primary English instruction. According to the data, 35.71% (*n* = 15) of the teachers utilize the textbook published by People’s Education Press, 42.86% (*n* = 18) adopt the textbook published by Hunan Education Press, and 21.43% (*n* = 9) employ other textbooks. When asked about their previous usage of English textbooks, 61.9% (*n* = 26) of the teachers have utilized textbooks from People’s Education Press, while 35.71% (*n* = 15) have used textbooks from Hunan Education Press; additionally, 28.57% (*n* = 12) have employed other textbooks.

Regarding course management, there are variations in the teaching responsibilities among teachers. 66.67% (*n* = 28) of the instructors dedicate 11 or more hours per week to English instruction, while 33.33% (*n* = 14) have a weekly class load of 10 h or less. Additionally, out of all the teachers, 42.86% (*n* = 18) teach exclusively one grade level, whereas the remaining 57.14% (*n* = 24) conduct English classes for two to six different grades. Notably, half of the teachers (50%) engage in instructing English across two distinct grade levels.

In terms of course participants, there is also variation in the amount of time students invest in learning English. Based on the data, 80.94% (*n* = 327) of students reported studying English for less than one hour per day, while 18.32% (*n* = 74) dedicated two to three hours daily to their English studies. Among the students, 37.38% (*n* = 151) had fewer than two weekly English classes, whereas 62.62% (*n* = 253) attended more than three classes per week; within this group, 16.34% (*n* = 66) had over five English classes each week.

Additionally, process evaluation aims to assess the identification of issues in course plan implementation and provide feedback for enhancing the course plan. The focus of process evaluation lies in the utilization of teaching resources, selection of teaching methods, and organization of on-site teaching (Xu and Liao, 2022). It encompasses the entire teaching implementation process and offers real-time feedback to promote curriculum development (Xu and Liao, 2022). According to Marxist theory on all-round development, emphasis should be placed on cultivating students’ psychological quality, social quality, ability quality, etc. (Shi, 2002). Therefore, the teaching process plays a crucial role in achieving student development. The theory of sustainable development also highlights the need to foster education’s sustainable development across content, means, and other aspects of instruction. The PEE process evaluation conducted in this study covers various aspects including course content (course difficulty and feedback), course delivery (teaching methods and approaches), as well as course learning (student participation and learning strategies). This comprehensive assessment helps us gain insights into teachers’ instructional modes and methods while considering students’ engagement throughout their learning journey.

In terms of the course content in the process evaluation, teaching difficulties arise from various sources including the school, teachers, students, curriculum, and environment. Table 4 provides a summary of the teaching challenges associated with these factors. Teachers emphasize that for effective English instruction, attention must be given to several key aspects involving themselves as educators, their students, and the English classroom. From a teacher’s perspective, it is crucial to focus on enhancing their own professional competence while also strengthening pedagogical reforms and classroom management techniques as well as fostering positive teacher-student relationships. Currently, students are required to actively engage in learning to overcome any obstacles they may encounter by improving their pronunciation skills and developing confidence in self-expression. Furthermore, within English classrooms there is a need to enhance knowledge interpretation design and facilitate practical application of acquired knowledge.

**Table 4:** Teaching difficulties.

School	Neglecting English classes and treating them as side courses; insufficient teaching equipment and insufficient teaching resources; the teaching facilities in rural schools are relatively backward, and the teaching and learning of teachers and students have limitations
Teachers	How to increase students’ interest in learning English and how to achieve efficient classroom teaching; the teaching effectiveness needs to be improved
Students	Lack confidence and are prone to forgetting words
Curriculum	Insufficient time for teaching
Environment	Lack of language environment

**Figure 4:**
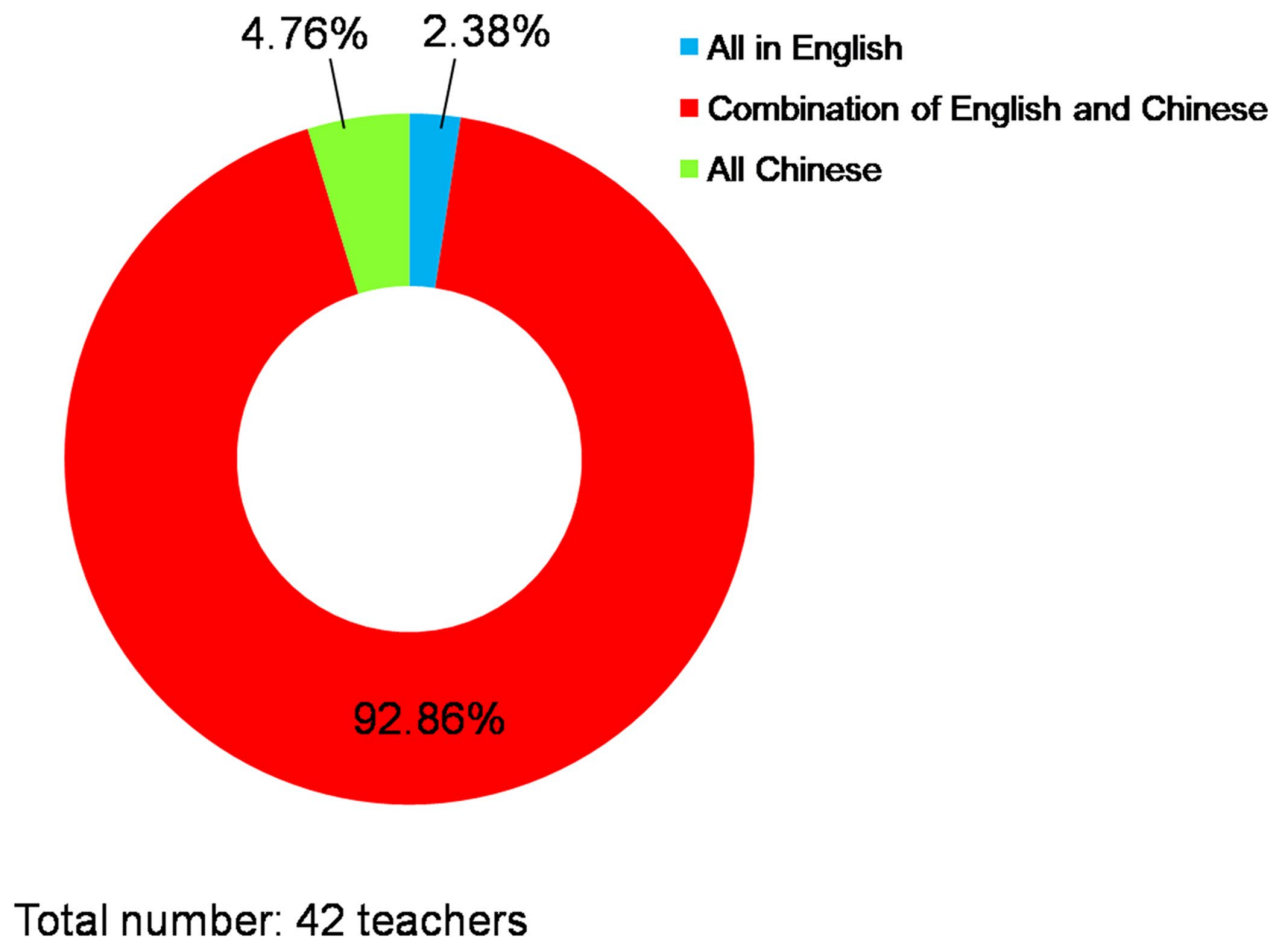
The main language used by teachers for teaching.

**Figure 5:**
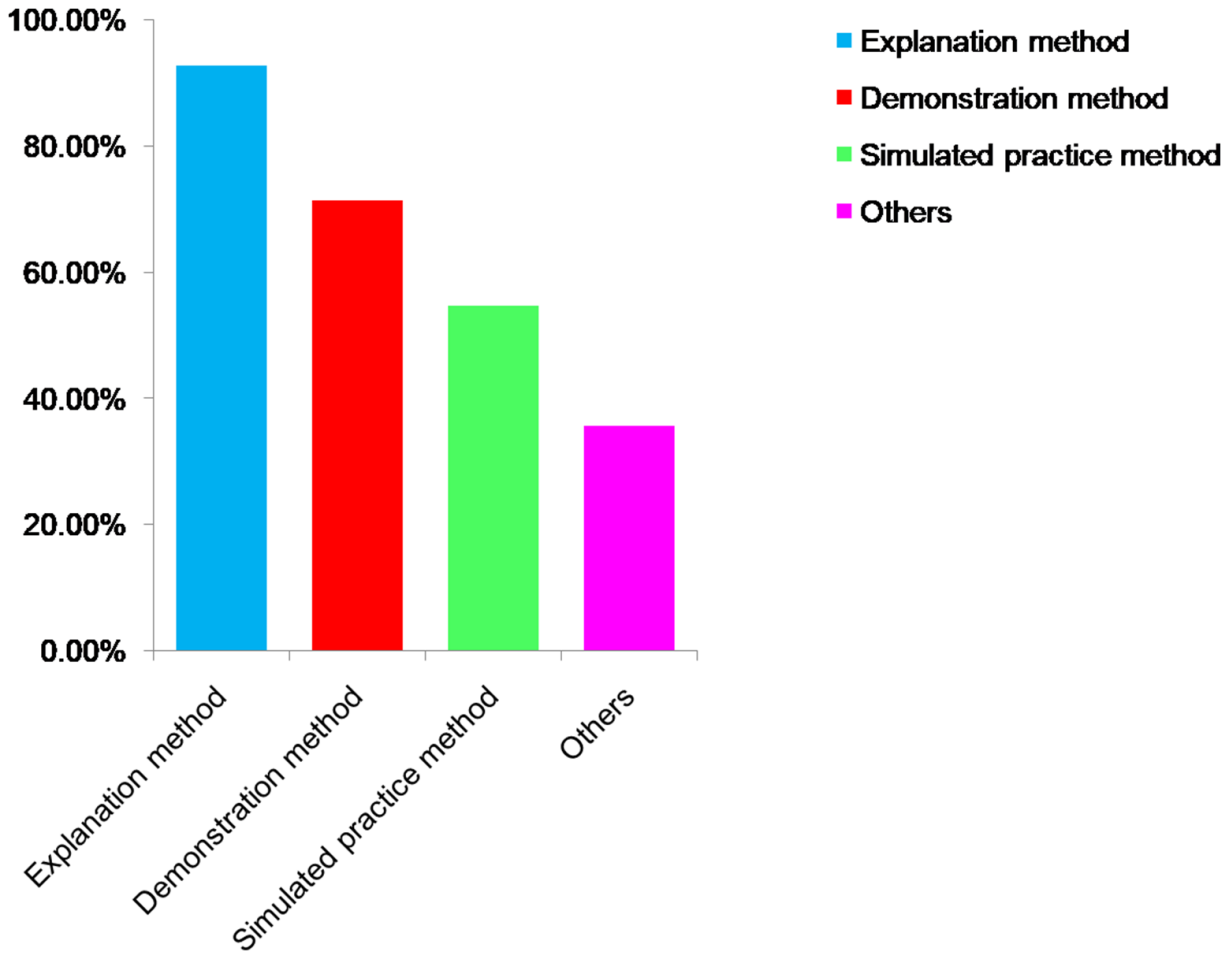
The methods used by teachers for teaching.

Respecting the course instruction, the predominant language employed by instructors was a blend of Chinese and English (see Figure 4), while the primary teaching methods remained explanation and demonstration (As depicted in Figure 5). Additionally, 78.57% (*n* = 33) of teachers incorporated English word cards into their teaching practices, whereas English phonetic cards were used by 42.86% (*n* = 18) of teachers who employed courseware. Concerning course delivery, frequent communication with teachers occurred among 29.21% (*n* = 118) of students, occasional communication among 37.87% (*n* = 153), while little to no communication in English was reported by 32.92% (*n* = 133) of students.

Touching course learning, the overwhelming majority of students resorted to conventional learning approaches, such as rote memorization of vocabulary (80.2%, *n* = 324), oral reading (74.5%, *n* = 301), and textual memorization (63.37%, *n* = 256). Subsequently, a considerable number engaged in listening to English songs (29.95%, *n* = 121) and watching English movies (22.52%, *n* = 91). Additionally, some students indicated that they enhanced their language skills through extracurricular book reading (20.05%, *n* = 81) and audio-based English practice sessions (13.12%, *n* = 53).

In the final assessment of product evaluation, this research places emphasis on evaluating the outcomes and effectiveness of promoting and verifying goal attainment (Xu and Liao, 2022). The purpose of product evaluation is to assess the efficacy of instruction, students’ learning outcomes and satisfaction, as well as identify areas for improvement through teachers’ and students’ reflections on teaching and learning. Sustainable development underscores practicality in evaluation by addressing problems and striving for improvements to achieve sustainable educational practices. Product evaluation aids in identifying teaching-related issues, thereby fostering sustainable development in English education. This study’s product evaluation encompasses learning effectiveness (skill-based goals, knowledge-based goals, emotional objectives), student satisfaction (course implementation), teacher professional growth and reflection (teaching reflection), as well as student progress and self-reflection (learning reflection).

In terms of learning effectiveness, the majority of students demonstrate proficiency in keeping pace with the teacher’s instructional progress and comprehending the teaching content. Among them, 22.03% (*n* = 89) of students reported a complete understanding of the material presented by the teacher, while 60.89% (*n* = 246) believed they grasped most of the lesson content. Additionally, 76.19% (*n* = 32) of teachers expressed confidence in their students’ ability to effectively acquire English knowledge or achieve mastery, with 85.71% (*n* = 36) acknowledging positive outcomes from their English learning endeavors. Furthermore, there was a general preference for attending English classes among 27.23% (*n* = 110) of respondents.

In relation to student satisfaction, students exhibit a positive inclination towards English learning, with the majority displaying willingness to engage in English language acquisition and communication with both educators and peers. Based on the gathered data, it was found that 78.71% (*n* = 318) of students perceived English classes as non-burdensome for their educational journey. Furthermore, 65.59% (*n* = 265) expressed eagerness to participate in English classes, while an overwhelming majority of 83.17% (*n* = 336) demonstrated readiness to interact in English with teachers and classmates during instructional sessions.

Considering teacher development and reflection, the surveyed teachers summarized the impact of teachers on students, the advantages and disadvantages of students’ English learning, the issues that arise in PEE, and their suggestions. Among the 42 surveyed teachers, a majority of 78.57% (*n* = 33) believed that integrating Chinese and English was an effective teaching method, while only a minority of 16.67% (*n* = 7) agreed that full English instruction was effective. Regarding their own competence in teaching, an overwhelming majority of 83.33% (*n* = 35) stated they were fully proficient, with only a small percentage of 16.67% (*n* = 7) acknowledging slight difficulties in teaching. Furthermore, nearly all teachers at a rate of 97.62% (*n* = 41) believed that student pronunciation is greatly influenced by their instructors; thus highlighting the importance for primary school students to learn English from teachers with proper pronunciation standards. Additionally, it is worth noting that out of all participating educators—approximately 90.48% (*n* = 38) expressed confidence in effectively completing lesson preparation as well as other instructional tasks assigned to them. The surveyed teachers also discussed children’s natural characteristics and learning challenges. Table 5 lists the advantages and disadvantages of primary school students learning English separately.

**Table 5:** Advantages and disadvantages of primary school students learning English.

Advantages	Full of curiosity; interested in learning English; good memory and obedience; best time for students to learn English; dare to speak up; good at imitating
Disadvantages	Fear of difficulties; lack self-discipline; inaccurate pronunciation; lack strong self-awareness; prone to setbacks when faced with difficult teaching content

The teachers also summarized the issues pertaining to equipment, faculty, students, and other aspects of PEE. They provided targeted suggestions as well. (Table 6 presents feedback from teachers on various matters such as faculty, students, facilities, schools, parents, classrooms, exams, courses, and resources along with corresponding recommendations).

**Table 6:** Problems and suggestions in PEE.

Categories	Problems
Teachers	Lack of professional teachers; heavy teaching tasks; high teacher mobility
Student	Difficulties in learning English
Facility	Lack of voice room
School	Insufficient emphasis
Parents	Unable to guide students in English learning
Classroom	Too little practice and insufficient teaching time
Exam	Difficult exam content
Course	Treat English classes as a subject to be tested
Resources	Insufficient teaching resources

Categories	Suggestions
Teacher	Hire professional teachers; reduce teaching tasks; hire foreign teachers
Students	Practice pronunciation; strengthen oral communication
Facility	Add a voice room
School	Increase emphasis on English; improve the status of primary school English teachers
Parents	Cooperate with teachers to cultivate students
Classroom	Increase teaching hours (for schools with only two English classes)
Exam	Reduce English exams
Course	Create an English language environment
Resources	Improve educational resource equipment

The results showed that 29.46% (*n* = 119) of the participants expressed confidence in their English learning and perceived themselves as proficient. A majority of the students (55.69%, *n* = 225) considered their English skills to be average, while a smaller proportion (12.62%, *n* = 51) believed their English learning was inadequate, and a minority (2.23%, *n* = 9) expressed confusion.

The primary challenge encountered by students in the process of learning English was their inability to retain vocabulary (61.14%, *n* = 247), along with difficulties arising from Chinese pinyin confusion (31.19%, *n* = 126). Additionally, they faced obstacles in comprehending the teacher’s instructions (25%, *n* = 101) and had limited time for English language acquisition (11.63%, *n* = 47). Furthermore, a subset of students, comprising 16.09% (*n* = 65), reported encountering other impediments to their learning journey.

### 3.2 Research question two was concerned with the deficiencies and causes of PEE in the Hunan Province

Although research suggests that teachers have enhanced their focus on English, improved their quality, diversified teaching methods and equipment, and heightened students’ interest in learning as well as their proficiency and satisfaction with English classes, there are still some areas for improvement.

The primary issue lies in the potential for educational inequality resulting from disparities in learning foundations stemming from family values and economic conditions. Since the implementation of PEE in 2001, Chinese leaders have expressed concerns regarding policy feasibility. For instance, Hu (2005) noted that China lacks the necessary conditions to develop a foreign language program at the primary school level overall. Liu (2001) raised questions about English curriculum standards, new teaching materials, and other related factors. They recognized that deficits in primary English education development were due to teacher shortages, inadequate teaching facilities, subpar curriculum standards and materials, as well as insufficiently qualified English teachers (Hu, 2007). In light of these issues, they recommended measures such as “accelerate the development of primary school English teachers,” “arrange a review of currently available teaching materials,” and so forth (Hu, 2007). Compared to the government’s policies, Chinese parents are concerned about their children not being exposed to English at an early age, in contrast to the government’s concerns regarding PEE. Some traditional Chinese beliefs emphasize the importance of learning foreign languages early on and believe that the longer one learns the better (Gui, 1987). Observing other children enrolling in training classes, many Chinese parents are also eager to send their own children for such classes. They worry that their children will fall behind from the very beginning (“children will lose at the starting line,” as per a traditional Chinese saying). Not only do certain primary schools, especially key elementary schools, prioritize English language education but major middle schools also consider it a specialty subject, providing students with a significant advantage when studying at prestigious institutions. Currently, China has a population of 1.4 billion (2023). In this developing country context where some students reside in economically prosperous areas while others live in remote and impoverished mountainous regions, there exists a substantial disparity in family economic conditions which poses as a major obstacle to students’ development of English proficiency. It is within this policy and environmental framework that students commence their English language learning journey.

**Figure 6:**
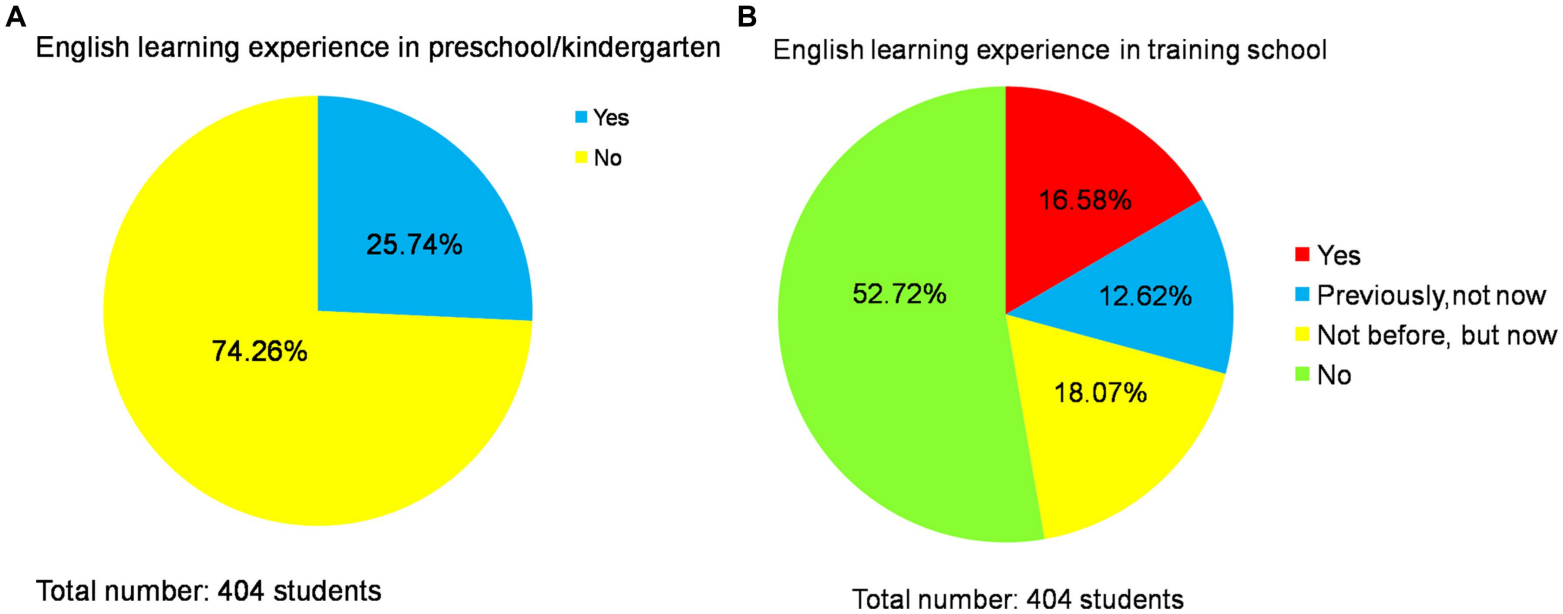
The differences in learning experiences in kindergarten, preschool, and training schools.

However, students’ learning experiences have demonstrated a discernible inclination towards educational inequality. Lu (2003) previously indicated that certain rural schools encounter difficulties in implementing policies due to various constraining factors; thus, as mentioned by Hu (2007), the persistence of educational inequality remains evident. This consequently results in unequal access to English education at the primary school level, thereby perpetuating educational disparities among children (Hu, 2007). As depicted in Figure 6, a significant proportion of students have engaged in training courses or acquired prior experience in learning English before commencing formal schooling. Specifically, 25.74% (*n* = 104) of students had studied English during their time in kindergarten or preschool, while the remaining 74.26% (*n* = 300) had not done so; furthermore, within this group who did not study English beforehand and now do so through training institutions accounts for 18.07% (*n* = 73), whereas those who previously studied English but no longer do comprise 12.62% (*n* = 51). Notably, there is also a substantial portion of students—52.72% (*n* = 213)—who have neither participated in any training courses nor received pre-primary English education whatsoever. These divergent learning experiences contribute to an inherent disparity within the realm of English education and subsequently give rise to discrepancies based on family economic circumstances and perceived importance attached to language acquisition during early childhood development stages. Figure 6 showcases the differences in learning experiences in kindergarten, preschool, and training schools.

The second issue of educational imbalance resulting from inadequate implementation of government policies remains prevalent. Since 2001, the Ministry of Education in China has issued “Guiding Opinions on Actively Promoting the Offering of English Courses in Primary Schools,” which clearly stipulated that English courses would gradually be offered in urban and county primary schools nationwide starting in autumn 2001, while township primary schools would offer them beginning autumn 2002. Typically, English classes are taught to third-grade students (Ministry of Education, 2001). As early as 2001, Lianning Li stated that the reason for learning English in the third grade of primary school was to prevent children from confusing Chinese pinyin and English letters. He also mentioned that, in some places where English letters are not taught, English teaching can start in the first grade of elementary school. However, in terms of the survey data on English teaching in primary schools, there is also an occurrence of educational imbalance resulting from the implementation of policies by schools. Firstly, 11.14% (*n* = 45) of students commenced learning English in the first grade, while 5.69% (*n* = 23) began in the second grade and a majority of 74.75% (*n* = 302) started in accordance with national regulations during their third-grade year; only a small percentage of students, namely 4.7% (*n* = 19), initiated English education in the fourth grade. The proportion further decreased to merely 0.99% (*n* = 4) and 2.72% (*n* = 11) for fifth and sixth graders, respectively, who were exposed to English instruction at that stage.

**Figure 7:**
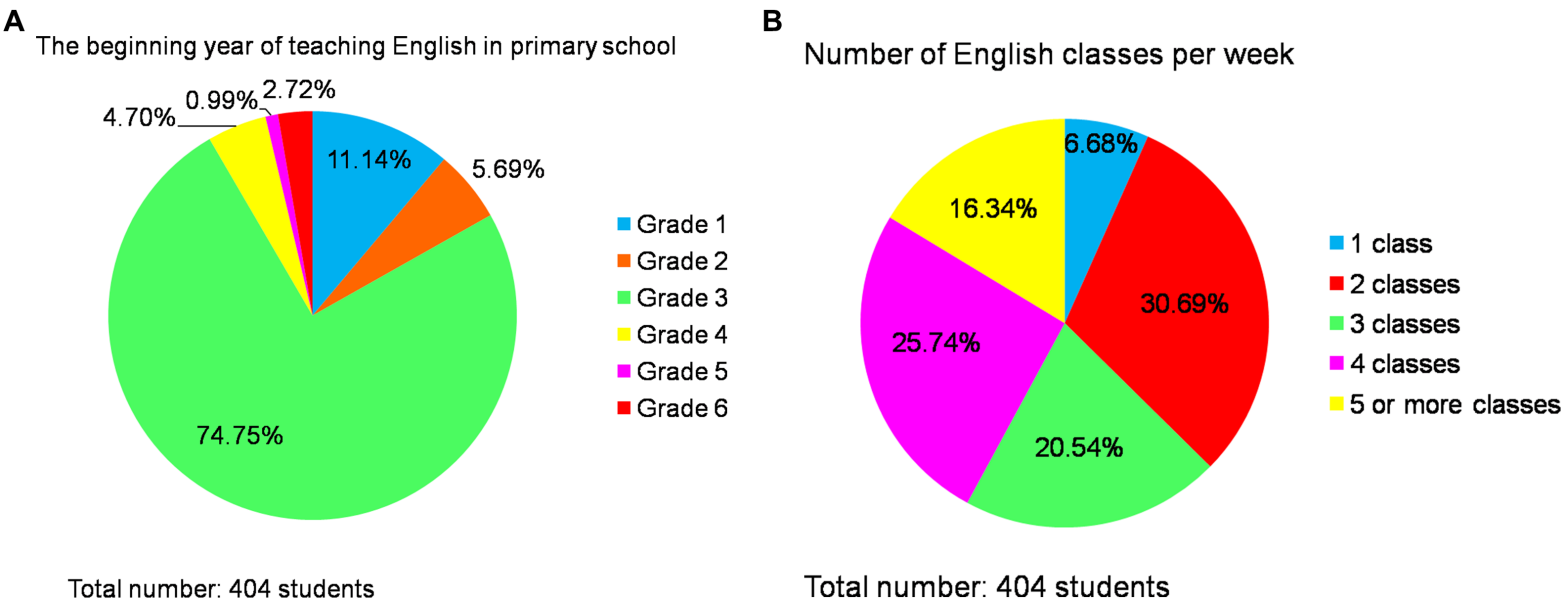
The grade when primary school started learning English and the number of English weekly classes.

Secondly, there was significant variation observed among different schools regarding weekly class hours dedicated to English instruction: approximately one-third or specifically speaking, 30.69% (*n* = 124), had two classes per week which accounted for the largest share; followed by four weekly class hours (25.74%, *n* = 104) and three weekly class hours (20.54%, *n* = 83). There were also cases where students received only one class per week (6.68%, *n* = 27), as well as those fortunate enough to have five classes per week (16.34%, *n* = 66). Figure 7 illustrates both when primary school students commence learning English and how many weekly classes they receive for this subject matter. The national policy emphasizes equitable development within English education; however, the allocation of class hours varies across different schools due to incomplete implementation by primary schools located in various regions.

The third issue is that the disparities in school teaching facilities and the number of English teachers can indicate an inequitable distribution of educational resources. Among the 42 surveyed teachers, a majority of 76.19% (*n* = 32) reported a shortage of less than three teachers in their respective schools. However, there is a significant disparity in the number of English teachers across other schools where these educators are located, exceeding four individuals, which highlights a deficiency in English language human resources within primary education institutions. Furthermore, this study revealed that only a mere 9.52 percent (*n* = 4) of the investigated teachers had access to voice rooms while variations were observed among different schools regarding other teaching equipment, thereby indicating an incomplete allocation of material resources.

The factors contributing to such uneven resource distribution stem from regional aspects, ideological influences as well as management issues including insufficient attention and funding problems. The economy, culture, and social environment of remote mountainous areas such as Zhangjiajie, Huaihua, and some rural areas differ significantly from those of developed urban centers. These differences have a direct impact on resource allocation. In many mountainous regions, there is a lag in economic and educational development, resulting in a heavy reliance on government financial support for the construction of school facilities. Consequently, funding sources become limited. Moreover, when it comes to teaching staff, numerous college students hold outdated employment beliefs that favor staying in urban areas rather than working in rural regions. This mindset contributes to an imbalance in English teaching personnel and creates disparities in staffing levels. Furthermore, school administrators often overlook issues related to resource allocation and teacher shortages which further exacerbate the imbalances between human resources and material resources.

Ultimately, the disparities in educational resources, the commencement of English language acquisition, and pedagogical approaches between urban and rural areas are substantial. The pronunciation of students in urban and rural areas is influenced by teachers to varying extents. From the perspective of educators, there exist disparities between rural and urban areas in terms of the extent to which students’ pronunciation is influenced by instructors and the utilization of English language laboratories (LABS) during English classes. Table 7 illustrates the impact of teachers on students’ pronunciation across various regions.

**Table 7:** The proportion of students’ pronunciation affected by teachers in different regions.

The proportion of students’ pronunciation influenced by teachers	Urban area	Rural area
Not too much	0.053	0.000
Relatively great significance	0.263	0.609
Great significance	0.684	0.391

**Figure 8:**
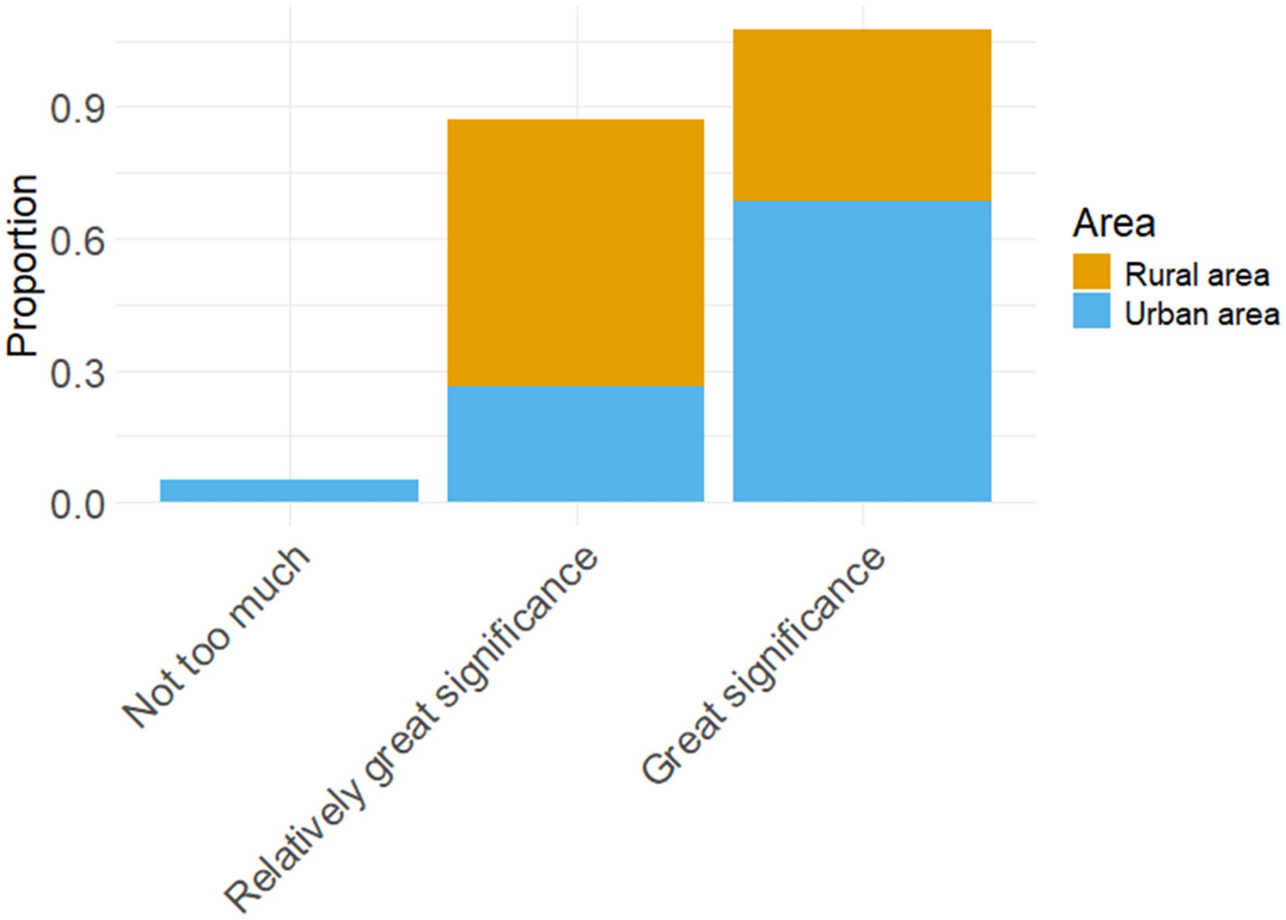
Stacked bar chart of the proportion of students’ pronunciation affected by teachers in different regions.

The contingency table above illustrates the correlation between teachers and students’ pronunciation, while categorizing them based on their geographical location (urban versus rural). Specifically, this variable is divided into three levels: not too much, relatively great significance, great significance. The contingency table cross-tabulates these two dimensions-geographical location (urban versus rural) and rating level-to demonstrate the distribution of each rating level across different geographical locations. Figure 8 presents a stacked bar chart illustrating the variation in teachers’ impact on students’ pronunciation across different regions.

The contingency table and stacked bar chart above clearly demonstrate discernible disparities in the distribution of students’ pronunciation influenced by teachers across different regions. Overall, both urban and rural areas exhibit a higher proportion of students greatly impacted by their teachers’ pronunciation, with values of 0.684 and 0.391, respectively. This indicates that in most instances, teachers have a significant influence on their students’ pronunciation. However, there exists a notable discrepancy in the degree of impact between urban and rural areas. In urban settings, the proportion of students whose pronunciation is influenced by teachers surpasses that in rural areas significantly, reaching 0.684. This phenomenon can be attributed to several factors including the relatively abundant educational resources available in cities, overall high quality of teaching staff, as well as the influence exerted by urban cultural environments on language learning among students. Additionally, urban learners are likely to be exposed to diverse linguistic surroundings which render them more susceptible to adopting their teacher’s pronunciation. Conversely, within rural regions where educational resources are comparatively scarce and variations exist in teacher proficiency levels regarding pronunciation skills along with a relatively homogeneous language environment; it is observed that while the highest proportion (0.609) still reflects an influence from teachers on student’s pronunciation albeit not classified as “great.” These circumstances may limit how extensively teachers can shape their students’ pronunciations. It is noteworthy that in rural areas, the proportion of students’ pronunciation being minimally influenced by teachers is 0, which could be attributed to either the limited sample size or unique regional circumstances. Nevertheless, this finding underscores the need for increased attention towards education in rural areas, particularly focusing on enhancing teachers’ pronunciation and teaching quality.

The disparity in the impact of teachers’ pronunciation on students’ speech across different geographical environments becomes apparent. Urban students are more susceptible to significant influence from their teachers’ pronunciation, whereas rural students may experience relatively less impact. This discrepancy can be attributed to various factors such as educational resources, teacher competence, and language environment. Future studies should delve into exploring how these factors affect the extent to which teachers influence students’ pronunciation and propose corresponding educational strategies to optimize learning outcomes.

In this study, Fisher’s Exact Test is further employed for statistical analysis of Count Data. This nonparametric test examines independence between two categorical variables when dealing with small sample sizes or low expected frequencies. Based on the results obtained from this test, it reveals a *p*-value of 0.0416 indicating statistical significance at a threshold level of 0.05; thus providing sufficient evidence to reject the notion that both categorical variables are independent from each other. Through Fisher’s exact test, we establish a robust statistical foundation supporting further exploration into potential relationships between these variables.

**Figure 9:**
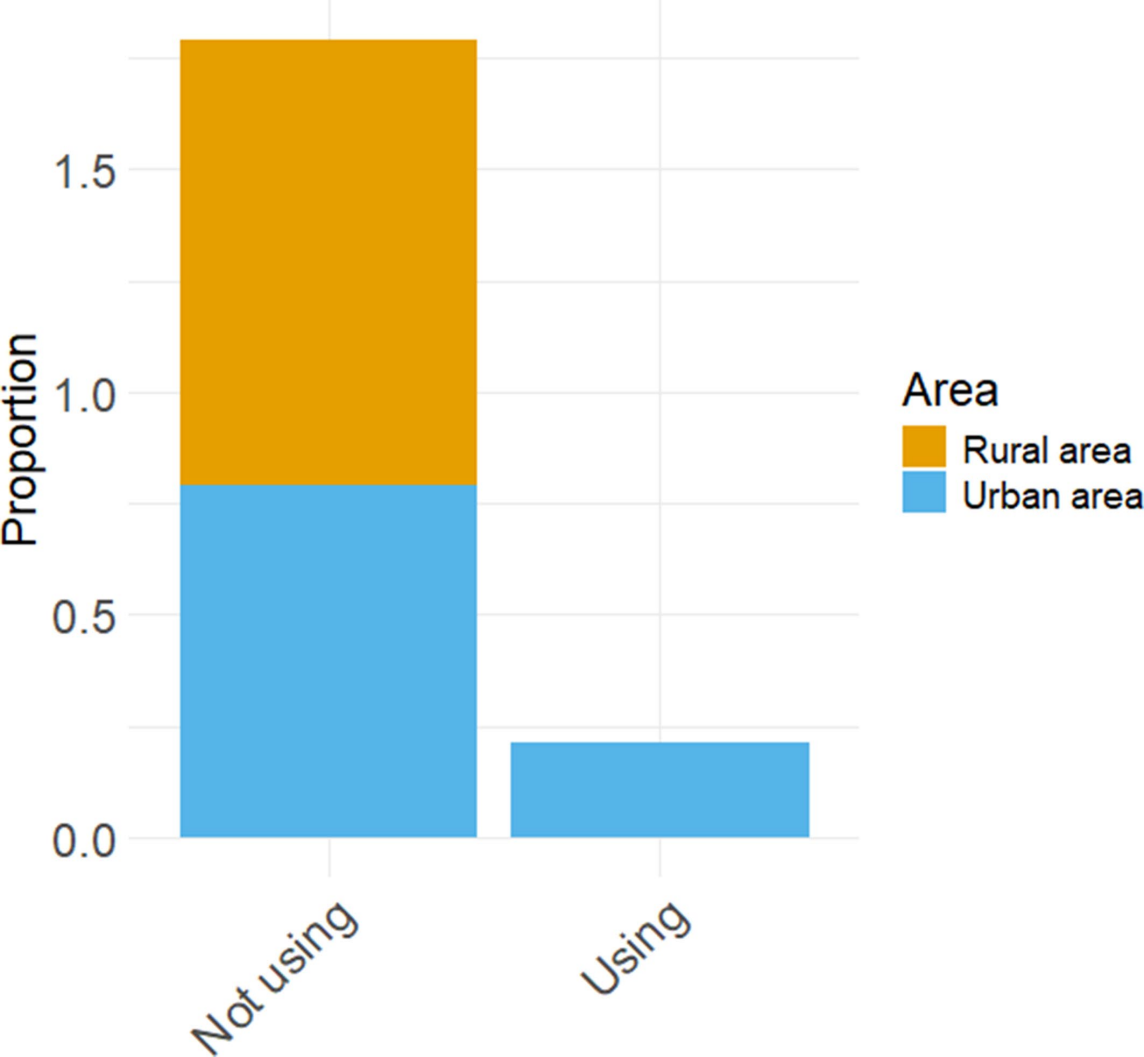
Stacking bar chart of the proportion of language LABS used by teachers in different regions in English classes.

The degree to which students’ pronunciation is influenced by teachers varies significantly, and there is also a notable disparity in the utilization of language laboratories between rural and urban areas. The utilization of language laboratories in English classes is clearly demonstrated by Table 8 and Figure 9, illustrating the practices employed by teachers in two distinct regions, namely urban and rural areas. Table 8 illustrates the distribution of language LABS utilization among teachers in various regions for English classes.

**Table 8:** The proportion of teachers in different regions using language LABS in English classes.

Percentage of teachers using the language lab in English classes	Urban area	Rural area
Non-use	0.789	1
Use	0.211	0

On the whole, there exists a significant disparity between urban and rural educators in their utilization of language laboratories (LABS) within English classes. In urban areas, the percentage of teachers who refrain from employing language LABS stands at 0.789, while only 0.211 of teachers opt to integrate them into their teaching practices. This indicates that despite some instructors choosing to incorporate language labs into English lessons in cities, the majority still exhibit a preference for abstaining from such usage. Conversely, the situation contrasts entirely in rural regions where all educators abstain from utilizing language LABS, accounting for a complete 100 percent. This notable regional discrepancy may reflect variations in educational resource allocation, teaching conditions as well as teachers’ willingness and proficiency in adopting novel technologies across different locales.

The breakdown table and stacked bar chart effectively illustrate the distribution of teachers using language rooms in English classes across different regions, highlighting significant disparities between urban and rural areas as well as the utilization patterns. This analysis will facilitate educators and policymakers in comprehending the current state of educational resource allocation, enabling them to formulate more rational policies that promote equitable distribution of resources and enhance teaching quality. Additionally, it suggests the need for further investigation and discussion on how to enhance teachers’ willingness and ability to utilize new technology, thereby maximizing the potential of modern educational technology for advancing education and instruction. Based on the results of independence testing, Fisher’s exact test yielded a *p*-value of 0.03463. As this *p*-value is less than 0.05, we have sufficient evidence to reject the null hypothesis that these two categorical variables are independent. The statistical correlation identified through Fisher’s exact test provides a robust foundation for exploring potential relationships between these variables.

In terms of students, the grade at which English is initiated and the level of English communication between teachers and students in English classes exhibit significant variations among students.

The distribution of different grades at which primary school students commence English education in urban and rural areas is presented in Table 9. In urban areas, the highest proportion (0.63) is observed among third-grade students, followed by second-grade students (0.2) and first-grade students (0.11). The percentage of students in Grade 4, Grade 5, and Grade 6 does not exceed 0.03 each. Conversely, in rural areas, the highest proportion (0.81) is found among third-grade students, followed by first-grade students (0.07), and fourth-grade students (0.06). The percentage of students in Grade two, Grade five, and Grade six does not exceed 0.03. This discrepancy in distribution may reflect the influence of various factors such as educational resource allocation, school size, and population mobility across different regions.

**Table 9:** The proportion of primary school students who began to learn English.

The proportion of grades in which primary school students begin to learn English	Urban area	Rural area
Grade one	0.20	0.07
Grade two	0.11	0.03
Grade three	0.63	0.81
Grade four	0.03	0.06
Grade five	0.01	0.01
Grade six	0.01	0.03

**Figure 10:**
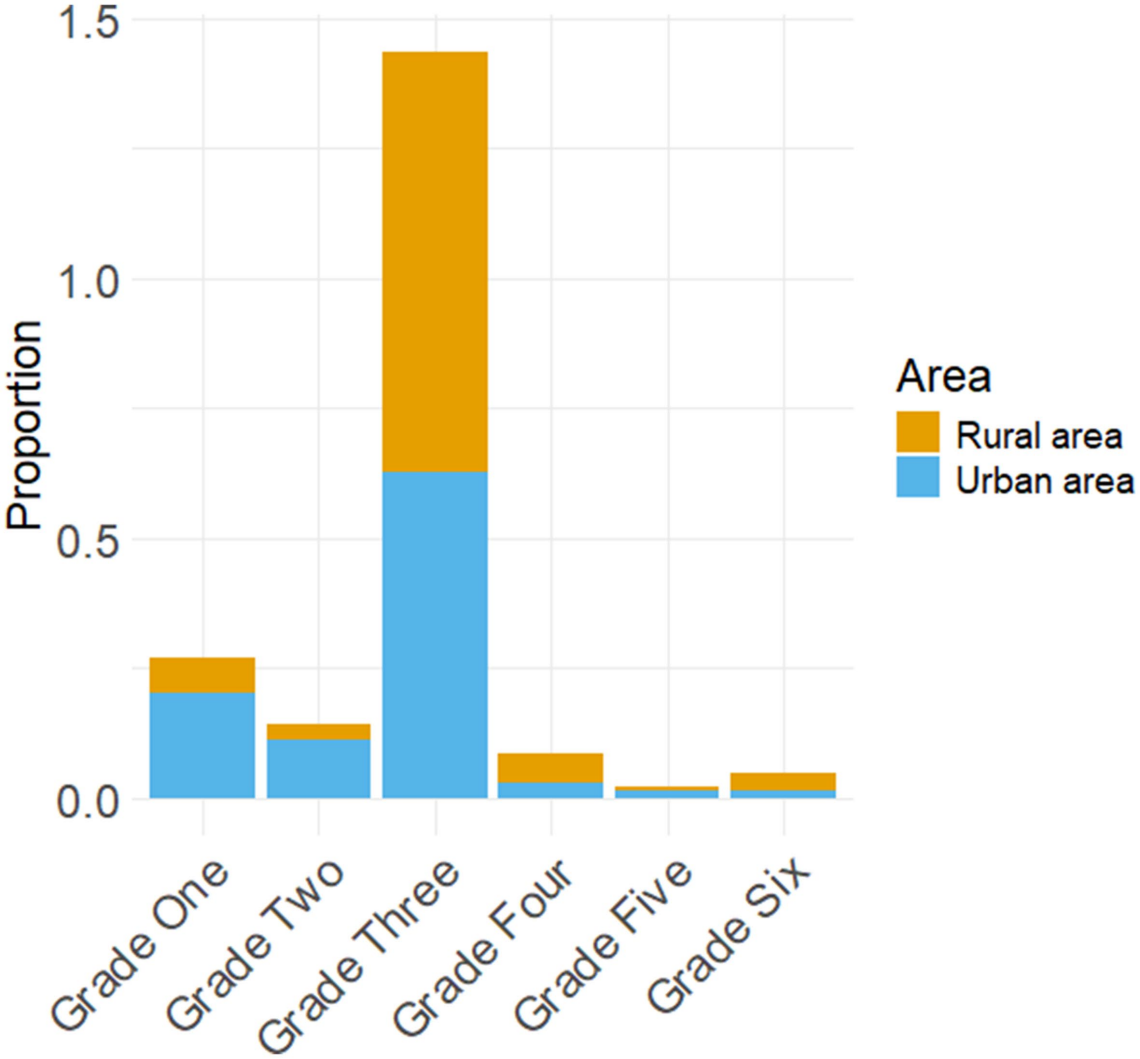
The grade in which pupils start to learn English.

From a regional perspective, Figure 10 illustrates a notable disparity in the distribution of English learning commencement among primary school students in urban and rural areas. In urban regions, there is a higher prevalence of grades 1, 2, and 3, while grades 4, 5, and 6 are less common. Conversely, in rural areas, there are a greater proportion of students in grades three and four compared to one; meanwhile, the proportion of students in grades two, five and six is lower. These regional discrepancies may reflect various factors such as educational demands, implementation of educational policies, and family economic conditions across different regions. To gain deeper insights into these variations and address them effectively, future studies should further investigate the impact of factors like resource allocation for education across diverse regions as well as the effectiveness of policy implementation on student distribution patterns. Additionally exploring the influence exerted by students’ family backgrounds would be crucial towards fostering balanced educational development.

The data presented in Table 10 illustrates the proportion of English communication between teachers and students in English classes, while also highlighting the disparities between urban and rural areas. Firstly, it is evident that regardless of geographical location, a majority of teachers engage in some level of English communication with their students during class. However, there are notable differences observed in the distribution of AC frequencies between these two regions. Table 10 presents the proportion of teachers and students engaging in English communication during English class.

**Table 10:** Proportion of teachers/students communicating in English in English class.

The proportion of teachers/students communicating in English in English class	Urban area	Rural area
No communication	0.142	0.104
Seldom communication	0.119	0.259
Occasional communication	0.425	0.356
Frequent communication	0.313	0.281

In urban areas, the proportion of occasional communication is the highest (0.425), indicating that in urban English classes, there is limited frequency of communication between teachers and students, with a significant amount of time dedicated to teaching or independent learning. Despite this, the proportion of frequent communication also reaches 0.313, suggesting that some teachers and students in urban areas are able to maintain relatively regular interaction. The proportions of no communication and minimal communication are relatively low at 0.142 and 0.119, respectively. In contrast, rural areas present a different scenario. Although occasional communication remains predominant at 0.356, it is lower compared to urban areas. Additionally, the proportion of individuals with minimal communication significantly increases in rural settings (reaching 0.259), surpassing those with no communication (at 0.104). This indicates that in rural English classes, there is comparatively less interaction between teachers and students which predominantly occurs at lower frequencies levels. The proportion of frequent communication in rural areas stands at 0.281—slightly lower than in urban areas; however considering the overall low level of interaction observed within rural communities, this figure still warrants attention.

**Figure 11:**
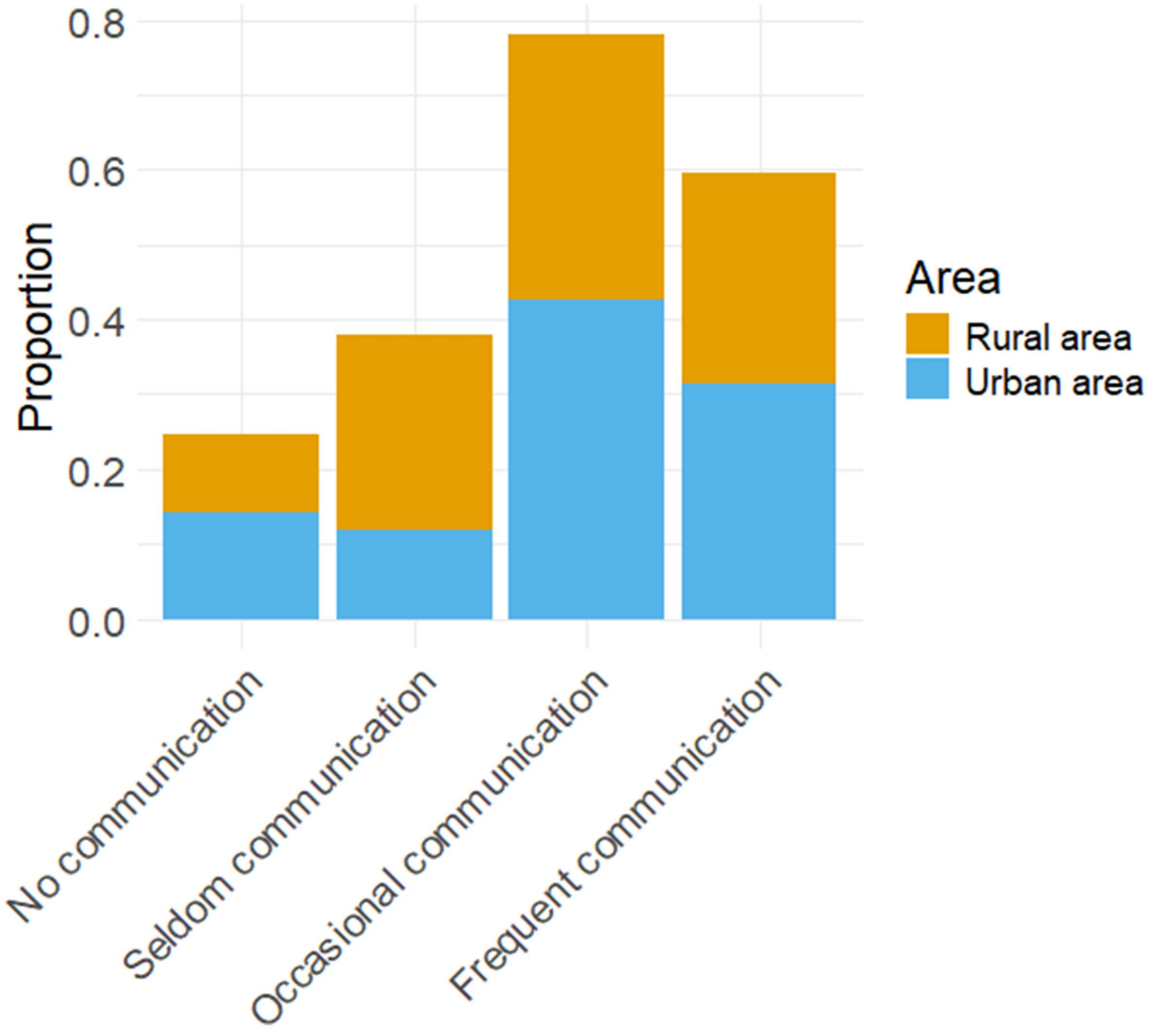
English communications between teachers and students in class.

To summarize, Table 10 and Figure 11 demonstrate a significant disparity in the proportion of English communication between educators and students in urban and rural areas. While the overall level of communication is commendable in urban regions, there remains a need to reinforce frequent interaction. Conversely, rural areas necessitate substantial improvement in communication frequency to enhance the quality of English instruction. Moving forward, educational authorities and institutions should devise targeted measures based on regional circumstances to ameliorate the communicative environment within English classes and augment students’ proficiency in practical language application.

### 3.3 Research question three focuses on the countermeasures to the problems

The educational inequality resulting from disparities in economic conditions and priorities, the educational imbalance caused by inadequate implementation of government policies, and the disparity in the allocation of educational resources due to geographical factors, conceptual factors, and issues with educational support, as well as variations in class start times, resources, and teaching methods contribute significantly to the disparities between rural and urban areas. Ultimately, these discrepancies impact students’ efficiency in acquiring knowledge and hinder sustainable development of English proficiency. These challenges ultimately stem from social factors, governmental actions, schools themselves, parents’ involvement, and students. This paper proposes solutions to address these issues through raising awareness levels among stakeholders involved in education systems while increasing investments and strengthening supervision mechanisms to narrow down the urban–rural divide.

First and foremost, it is crucial to enhance the awareness of sustainable development, bolster the teaching team’s capacity, and prioritize sustainable teaching. In order to advance the sustainable development of PEE, the Chinese government is fully committed to upholding its sustainability goals. For example, the Ministry of Education has issued the “Compulsory Education Curriculum Plan and Curriculum Standards (2022 Edition),” which puts forward the requirements for students, teachers, and curriculum. The requirements for the development of students’ core competencies, such as listening and speaking, have been clearly defined on the student side. In terms of curriculum optimization, there is an emphasis on establishing a connection between primary and secondary schools, updating practical curriculum content, improving the organization and presentation of curriculum materials, highlighting the interrelation of knowledge within and across disciplines, and proposing academic quality standards. The new curriculum standard also introduces fresh expectations for teachers by emphasizing their guidance in comprehending teaching depth and breadth. These policies are formulated to address challenges arising in PEE during this new era while providing future development guidelines for PEE. It is worth noting that the document emphasizes the importance of bridging primary and secondary schools to lay a foundation for continuous English learning among primary school students while reducing deficiencies in their English language acquisition.

According to research findings, the lack of awareness regarding the sustainable development of Primary English Education (PEE) stems from both parental and educational factors. Some parents prioritize enrolling their children in various English training programs prior to primary school, while others neglect English learning altogether. This discrepancy leads to challenges in bridging early childhood education with primary school English curriculum, highlighting a disparity in parental awareness towards sustainable development. Furthermore, there is reluctance among professional English teachers to teach in rural areas, indicating a lack of national sustainable development consciousness within PEE. Additionally, schools’ insufficient number of qualified English teachers and unequal distribution of resources and facilities are issues that require attention for the sustainable development of teaching staff and educational infrastructure within PEE. At the United Nations World Summit on Sustainable Development held in 2002, the emphasis was placed on educational reform, proposing to “enable students to acquire skills, perspectives, values, and knowledge for sustainable living in communities” (Qian, 2005). The sustainable development of students cannot be achieved without the support of schools, teachers, and parents, especially teachers. The International Implementation Plan for the United Nations Decade of Education for Sustainable Development (2005–2014) emphasizes strengthening teachers’ awareness of sustainable development, enhancing their communication, evaluation, education, and management abilities, improving teaching methods, and educating them about sustainable development education through pre-job and in-service training. It also utilizes high-quality teaching methods of sustainable development education to incorporate them into curriculum content to ensure the smooth implementation of sustainable development education (Qian, 2005). Therefore, it is necessary to enhance the sustainable development awareness of schools, teachers, and parents from both ideological and practical perspectives. For example, the education department can incorporate sustainable development into the school evaluation system, using sustainable development awareness as one of the indicators to assess the excellent quality of teachers (Zhang, 2002), improve the professional level of teachers in school through on-the-job training, hire sufficient professional teachers, and strive to build a teaching staff with sustainable development awareness; for teachers who are waiting for employment, they should change their mindset, establish a correct outlook on employment, and contribute to the needs of the motherland and the people; for the cultivation of parents’ sustainable awareness, local governments or schools can arrange sustainable development awareness education and social practice in a planned manner.

Secondly, it is a prerequisite to uniting with social forces to increase investment in education. In the specific goals of the “Education Decade” in the “International Implementation Plan,” it is mentioned to promote communication and interaction among relevant units of sustainable development education, provide space and opportunities for sustainable development, formulate strategies to strengthen sustainable development education, and continuously improve the teaching quality of sustainable development education (Qian, 2005). The Outline of the National Medium- and Long-Term Education Reform and Development Plan (2010–2020) also mentions the need to increase investment in education. Faced with the material differences in educational facilities among different schools, schools not only need to strive for government support but also seek social forces to promote the sustainable development of school educational resources. The government department takes the lead in supporting social organizations and individual funding, utilizing media to promote the accomplishments of funding, establishing a specialized education funding management department, and striving to raise education funding through multiple channels to create an optimal social funding environment (Li, 2010). Therefore, in order to facilitate the sustainable development of PEE and establish a development system that ensures both efficiency and fairness, primary schools should endeavor to mobilize more parents and society at large to guarantee the fundamental educational facilities and foster the enduring progress of PEE in terms of material resources.

Thirdly, it is imperative for the government to exercise supervision while schools collaborate in order to ensure the equilibrium of educational resources and promote educational equity. Educational equity serves as the cornerstone for fostering a harmonious society and ensuring social fairness. Whether at primary, middle, high school or university level, students are entitled to an equitable starting point in education. Furthermore, there should be fairness in the allocation of public education resources including per student average value of teaching equipment, per student average building area of school facilities, student–teacher ratio, as well as the educational structure and professional title distribution among full-time teachers (Wang, 2008). The government also bears the responsibility of safeguarding vulnerable groups by providing them with fundamental rights and opportunities for survival and development (Wang, 2008). The Outline of the National Medium- and Long-Term Education Reform and Development Plan (2010–2020) stipulates that education is the foundation for promoting national rejuvenation and social development and progress. It is imperative to promote the well-rounded development of compulsory education, ensuring universal access and obligation to education, while elevating the pursuit of educational equity as a fundamental national policy. Students should not only be granted admission but also provided with equal opportunities and conditions for receiving education, including equitable distribution of teachers, balanced infrastructure development, and fair allocation of educational resources. “The China Education Modernization 2035,” issued by the Central Committee of the Communist Party of China and the State Council, emphasizes “ensuring satisfactory education for all,” “prioritizing inclusivity,” “achieving high-quality and equitable compulsory education,” “establishing and enhancing academic standards across various disciplines in primary and secondary schools,” and “implementing scientifically planned curricula for universities, as well as primary and secondary schools.” This outline embodies the principles of educational fairness and equitable allocation of resources while progressively advancing primary education development. To achieve balanced development in education, it is essential to ensure that the educated enjoy equal treatment and promote their comprehensive development in the face of fair opportunities. Sustainable development education is the integration of sustainable development concepts into all aspects of learning in order to change people’s behavior and build a more sustainable and just society for all (Qian, 2005). Currently, the government is making great efforts to promote fair education and the balance of educational resources. To deeply implement the national “double reduction” policy, further reduce the burden of extracurricular training for primary and secondary school students, and bridge the gap between students in English learning, all extracurricular training institutions in the Hunan Province have been included in the unified management of the national extracurricular education and training supervision and service comprehensive platform. As of December 3, 2021, education administrative departments at all levels in the Hunan Province had cancelled the educational licenses of 1,280 illegal extracurricular training institutions (Sanxiang City Daily, 2021). According to Fa (2022) No. 30, six departments, including the Education Department of the Hunan Province, have standardized the management of non-disciplinary off-campus training institutions (Education Department of the Hunan Province, 2022). From this perspective, it can be seen that the education department is striving to narrow the uneven gap in education and overcome the problems in PEE, both in terms of policies and actions.

Finally, it is imperative for the state to narrow the urban–rural education gap through targeted policy support. In July 2010, the CPC Central Committee and the State Council issued the Outline of the National Medium- and Long-Term Education Reform and Development Plan (2010–2020), commonly known as the “Education Planning Outline,” which proposed establishing an integrated mechanism for compulsory education development in both cities and villages. This mechanism includes prioritizing rural areas in terms of financial allocation, school construction, and teacher distribution. According to surveys, significant disparities exist in terms of teacher qualifications, teaching quality, and school starting times. Therefore, it is crucial for the government to promote policy-level integration of urban and rural educational resources. Firstly, policies should be formulated to engage experts who can supervise teaching quality in both urban and rural areas while ensuring compliance with regulations regarding English class schedules in schools. Additionally, national policies supporting mobile teaching by urban and rural teachers can facilitate communication between them and alleviate the urban–rural education gap to a certain extent.

The disparities in weekly English class hours, variations in English teaching start times, challenges with school facility allocation, and deficiencies in professional teachers identified in this study present a series of obstacles to achieving educational equity for PEE. The education department bears the responsibility of regularly supervising, implementing preventive supervision and development measures, maintaining equal emphasis on supervision and support, and promoting fair and reasonable arrangements for English teaching across the province. Due to China’s high population density, the implementation of educational management is bound to encounter various issues. The education department has been proactive by introducing multiple policies to promote educational equity and balance educational resources. However, it is still necessary for government departments to further supervise the implementation of these policies in order to raise awareness of sustainable development among schools, teachers, and parents while striving for increased funding from society.

## 4 Conclusion

The present study has both theoretical and practical implications. The study presents a theoretical framework for assessing the quality of English education in primary schools, based on the PICC theory, thereby providing a solid foundation for evaluating the effectiveness of English instruction at this level. Furthermore, the research also summarizes the development progress and deficiencies in PEE, affirming that the education department has made adjustments to promote educational equity and resource balance and is striving to promote PEE. From the perspective of practical implications, research on the status quo of primary school English teacher education in the Hunan Province is conducive to promoting the development of PEE. First, the evaluation of educational quality from the perspectives of school, teachers, students, and curriculum provides data for teachers to conduct self-analysis and evaluation, which is beneficial for teachers to understand the problems in English education. In addition, teachers can also use data to conduct more accurate analyses and evaluations of education quality, helping them have a more comprehensive and accurate grasp of their own and group information, making it possible for teachers to implement individualized education according to their aptitude, which is conducive to the overall quality of PEE. Furthermore, education managers can comprehensively understand the quality of teachers’ education through data, cultivate and attract outstanding talents with targeted goals, enhance the strength of foreign language teachers, and improve the efficiency of internal management within the organization.

The present study covered a wide range of regions and schools; therefore, the main limitation of this study is the small sample size of teachers and the equitable distribution of teacher groups across rural and urban schools within the Hunan Province. Although the sample size of English teachers in primary schools in the Hunan Province is relatively small, these data are representative and can effectively reflect the challenges faced by teacher education. Specifically, 45.24 percent of the participants are from urban primary schools in the Hunan Province, while 54.76 percent come from rural areas. It is important to note that all participants hold permanent teaching positions and do not engage in administrative work, demonstrating their willingness to actively cooperate with the investigation. Besides, the research did not adequately consider the differences and correlations between urban primary school English education and rural primary school English education. Although the current research indicates minimal disparities between urban and rural areas in terms of teacher conditions, curriculum offerings, and student demographics, it is important to note that these survey findings are solely limited to the specific aspects examined within this study. The questionnaire content arrangement did not fully incorporate the classification based on CIPP theory. For instance, curriculum teaching evaluation could have been divided into aspects such as teaching attitude, teaching content, and teaching organization form. The future research should prioritize the sample questions of both rural and urban teachers and students, while placing additional emphasis on assessment classifications such as educators’ teaching attitudes and students’ English proficiency, in order to establish an integrated assessment system. Moreover, in order to facilitate an in-depth analysis of urban–rural disparities, differentiated assessments along with correlation analyses will be carried out while considering their respective characteristics.

## Data availability statement

The original contributions presented in the study are included in the article/supplementary material, further inquiries can be directed to the corresponding author.

## Ethics statement

Ethical approval was not required for the studies involving humans because this research did not relate with ethical problems. The studies were conducted in accordance with the local legislation and institutional requirements. Written informed consent for participation was not required from the participants or the participants’ legal guardians/next of kin in accordance with the national legislation and institutional requirements because this research did not contain any information about ethical problems. Written informed consent was obtained from the individual(s), and minor(s)’ legal guardian/next of kin, for the publication of any potentially identifiable images or data included in this article.

## Author contributions

XC: Conceptualization, Data curation, Formal analysis, Funding acquisition, Investigation, Methodology, Project administration, Resources, Supervision, Validation, Writing – original draft, Writing – review & editing. ZW: Writing – review & editing, Data curation.

## Footnotes

^1^Some teachers’ majors do not match their educational backgrounds, and this part is used to investigate whether the majors they learn are consistent with the majors they teach.

^2^The limited representation of students in lower grades (grades 1 to 3) has been emphasized by one reviewer who suggests focusing exclusively on students from grades 4 to 6. However, it should be noted that the sample size for grade one and grade two is relatively small due to most primary schools starting English classes after grade three, as supported by research findings. Considering the involvement of educational resources and policies, it is deemed necessary to retain this portion for further discussion. Please refer to Part 4 for detailed information.

^3^The indicator classification in this survey was constructed based on the indicators proposed by Yin (2019), see reference “Yin, W. (2019). Research on the construction of course evaluation system of college foreign language flipped classroom based on CIPP. *Journal of Ocean University of China*. 3, 121–128.”
